# Aminoglycerophospholipid flipping and P4-ATPases in *Toxoplasma gondii*

**DOI:** 10.1016/j.jbc.2021.100315

**Published:** 2021-01-21

**Authors:** Kai Chen, Özlem Günay-Esiyok, Melissa Klingeberg, Stephan Marquardt, Thomas Günther Pomorski, Nishith Gupta

**Affiliations:** 1 Department of Molecular Parasitology, Faculty of Life Sciences, Humboldt University, Berlin, Germany; 2 Department of Experimental Biophysics, Faculty of Life Sciences, Humboldt University, Berlin, Germany; 3 Department of Biological Sciences, Birla Institute of Technology and Science Pilani (BITS-P), Hyderabad, India

**Keywords:** NBD-lipid, phosphatidylserine, phosphatidylethanolamine, P4-ATPase, Lem3/Cdc50, 3′IT, 3′-insertional tagging, AID, auxin-inducible degron, BSA, bovine serum albumin, DHFR-TS, dihydrofolate reductase–thymidylate synthase, HFF, human foreskin fibroblast, HXGPRT, hypoxanthine-xanthine-guanine phosphoribosyltransferase, IAA, indole-3-acetic acid, NBD, nitrobenzoxadiazole, NEM, N-ethylmaleimide, PV, parasitophorous vacuole, PSD, phosphatidylserine decarboxylase, PtdCho, phosphatidylcholine, PtdEtn, phosphatidylethanolamine, PtdSer, phosphatidylserine, SM, sphingomyelin

## Abstract

Lipid flipping in the membrane bilayers is a widespread eukaryotic phenomenon that is catalyzed by assorted P4-ATPases. Its occurrence, mechanism, and importance in apicomplexan parasites have remained elusive, however. Here we show that *Toxoplasma gondii*, an obligate intracellular parasite with high clinical relevance, can salvage phosphatidylserine (PtdSer) and phosphatidylethanolamine (PtdEtn) but not phosphatidylcholine (PtdCho) probes from its milieu. Consistently, the drug analogs of PtdCho are broadly ineffective in the parasite culture. NBD-PtdSer imported to the parasite interior is decarboxylated to NBD-PtdEtn, while the latter is not methylated to yield PtdCho, which confirms the expression of PtdSer decarboxylase but a lack of PtdEtn methyltransferase activity and suggests a role of exogenous lipids in membrane biogenesis of *T. gondii*. Flow cytometric quantitation of NBD-probes endorsed the selectivity of phospholipid transport and revealed a dependence of the process on energy and protein. Accordingly, our further work identified five P4-ATPases (*Tg*P4-ATPase1-5), all of which harbor the signature residues and motifs required for phospholipid flipping. Of the four proteins expressed during the lytic cycle, *Tg*P4-ATPase1 is present in the apical plasmalemma; *Tg*P4-ATPase3 resides in the Golgi network along with its noncatalytic partner Ligand Effector Module 3 (*Tg*Lem3), whereas *Tg*P4-ATPase2 and *Tg*P4-ATPase5 localize in the plasmalemma as well as endo/cytomembranes. Last but not least, auxin-induced degradation of *Tg*P4-ATPase1-3 impaired the parasite growth in human host cells, disclosing their crucial roles during acute infection. In conclusion, we show selective translocation of PtdEtn and PtdSer at the parasite surface and provide the underlying mechanistic and physiological insights in a model eukaryotic pathogen.

*Toxoplasma gondii* is an obligate intracellular pathogen of the protozoan phylum Apicomplexa, which has a marked ability to infect, reproduce, and persist in almost all homeothermic organisms including humans. The parasite inflicts acute and chronic toxoplasmosis that is mostly asymptomatic in healthy individuals, but its opportunistic infection can be fatal in people with weak or compromised immunity (1). Tissue necrosis is caused by rapid asexual reproduction of its acute (tachyzoite) stage in a wide range of nucleated host cells. Tachyzoites undergo a reversible stage switching between infectious extracellular and replicative intracellular phases during the lytic cycle, which comprises the serial events of invasion, replication, and egress (2). After invasion, the parasite dwells in a parasitophorous vacuole (PV) that is originally derived from the host plasma membrane but profoundly modified to enable its asexual reproduction by endodyogeny (3, 4). The formation of progeny and concurrent expansion of the PV require extensive membrane biogenesis that is satisfied by *de novo* synthesis as well as salvage of lipids from the parasite environment (5, 6, 7, 8, 9, 10, 11, 12, 13, 14, 15).

The tachyzoite membranes consist of several phospholipids, such as phosphatidylcholine (PtdCho), phosphatidylethanolamine (PtdEtn), phosphatidylinositol, phosphatidylthreonine, phosphatidylserine (PtdSer), phosphatidic acid, ethanolamine phosphorylceramide, inositol phosphorylceramide and sphingomyelin (SM) (5, 8, 16, 17). As known in other eukaryotic cells (*e.g.*, yeast, mouse, human), phospholipids are asymmetrically distributed between the leaflets of the membrane bilayer, such as in the Golgi apparatus, endosome, and plasma membrane. PtdSer and PtdEtn are located primarily in the cytoplasmic leaflet, whereas PtdCho and sphingolipids are enriched on the exoplasmic side facing the extracellular space or organelle lumen (18, 19, 20, 21). Transbilayer distribution of lipids is established by a diverse set of ATP-dependent translocases, which normally include a catalytic α subunit (P4-ATPase) and a noncatalytic β subunit (Lem3/Cdc50). The coupling of α and β subunits is imperative for lipid flipping and proper subcellular localization (22, 23), although not all P4-ATPases require a partner protein.

P4-ATPases or flippases have so far only been reported in eukaryotes. These proteins have gained significant attention after recent seminal studies describing the structure and mechanism of human and yeast flippases (24, 25, 26). The human genome harbors 14 such proteins grouped into P4A and P4B classes (27). The genome of *Saccharomyces cerevisiae* on the other hand encodes five flippases known as *Drs2*, *Dnf1*, *Dnf2*, *Dnf3*, and *Neo1* (21). Among other functions, P4-ATPases are involved in organelle biogenesis, endocytic trafficking, phospholipid synthesis, apoptosis, and cellular aging (28, 29, 30, 31, 32, 33, 34, 35, 36). For instance, PtdSer- and PtdEtn-specific flippases ensure enrichment of these lipids on the cytoplasmic leaflet of specific organelle membranes and thereby promote the binding of cytoskeletal components, adapter proteins, or enzymes to the subcellular membranes, which are vital for signaling cascades, catalytic regulation, metabolism, vesicle trafficking, and cell motility (37, 38, 39). Evenly, certain P4-ATPases can transport lyso-PtdEtn and lyso-PtdCho (*e.g.*, *Sc*Dnf1/*Sc*Dnf2 in *S. cerevisiae*) and subsequently facilitate the regeneration of PtdEtn and PtdCho to fuel a rapid cell growth (30, 31).

Given the dynamic lipid and ionic milieu that apicomplexan pathogens encounter inside and outside their respective host cells, lipid flipping is anticipated to be important during the extracellular and intracellular parasitic stages. In accord, genomes of these parasites also harbor several putative P4-ATPases (www.EuPathDB.org); however, in notable disparity to their mammalian host cells, let alone other eukaryotes, little to none is known about phospholipid flipping in apicomplexan parasites. Here we deployed *T. gondii*, a model intracellular pathogen, to examine the occurrence, mechanisms, and importance of aminoglycerophospholipid flipping in its acutely infectious tachyzoite stage.

## Results

### Tachyzoites of *T. gondii* can selectively salvage PtdSer and PtdEtn

**Figure 1:**
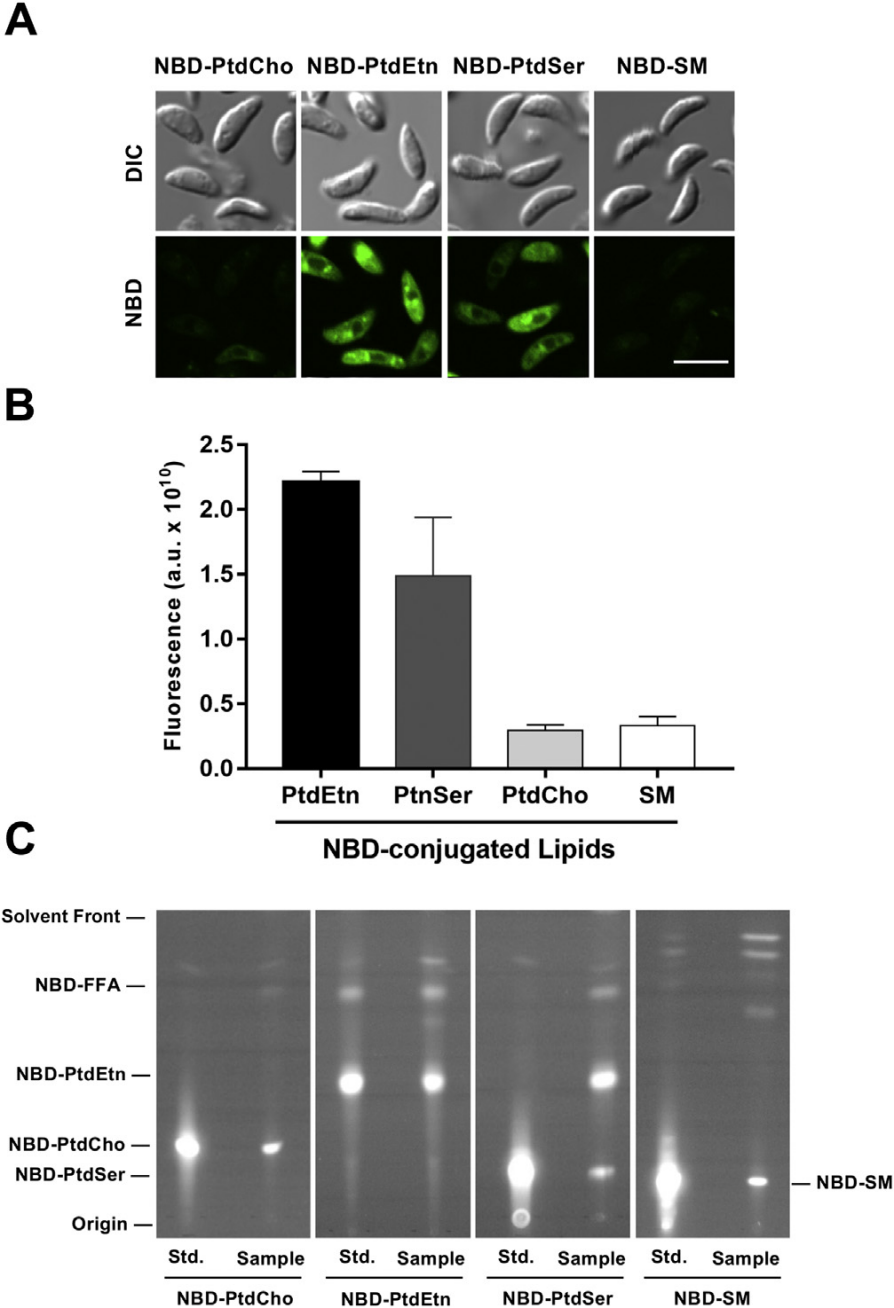
***Toxoplasma gondii* can salvage PtdSer and PtdEtn from the extracellular environment.** Tachyzoites were incubated with NBD-lipids (37 °C, 30 min), followed by microscopic imaging, flow cytometry, and thin-layer chromatography. *A*, imaging of parasites immediately after incubation with NBD-conjugated PtdEtn, PtdSer, PtdCho, or sphingomyelin. Cells were examined by fluorescence microscopy and differential interference contrast (DIC). The scale bar represents 10 μm. *B*, geometric mean fluorescence intensity of NBD-labeled tachyzoites, as determined by flow cytometry (n = 3; mean ± SE). *C*, TLC-resolved lipid profiles of parasites treated with indicated probes. Lipid samples were resolved onto a silica plate along with standards (Std.) and imaged for fluorescence. *NBD-FFA*, NBD-linked free fatty acids. NBD, nitrobenzoxadiazole; PtdCho, phosphatidylcholine; PtdEtn, phosphatidylethanolamine; PtdSer, phosphatidylserine.

To determine whether extracellular tachyzoites can import aminoglycerophospholipids, we incubated them with nitrobenzoxadiazole (NBD)-conjugated PtdEtn, PtdSer, PtdCho, and SM (negative control) (Fig. 1*A*). Fluorescence imaging of the probe-labeled parasites showed import of PtdSer and PtdEtn, as evident by the punctate intracellular NBD signal distributed throughout the parasite body. In contrast, the intracellular fluorescence of NBD-PtdCho was notably poor and comparable with the background fluorescence of NBD-SM (Fig. 1*A*). Quantification by flow cytometry confirmed the microscopy data, revealing a marked internalization of NBD-PtdEtn and NBD-PtdSer (Fig. 1*B*). Uptake of PtdEtn and PtdSer probes was four to eight times more than PtdCho as well as SM, and the relative import of specified probes was comparable with our microscopic observation. In extended work, we tested the drug analogues of PtdCho (miltefosine and edelfosine) in plaque assays to examine their effect on the parasite growth (Fig. S1). None of the two drugs exerted a notable effect as increasing doses (1–100 μM) resulted in only a modest impairment of the lytic cycle. A maximum of 45% inhibition was seen at the highest 100 μM concentration of drugs (100–200× more amount than NBD-probes used here), which echoes well with a poor import of NBD-PtdCho. In brief, the data disclosed a selective uptake of lipid analogues across the plasma membrane of tachyzoites.

### Internalized PtdSer is decarboxylated to PtdEtn, but the latter is not methylated to PtdCho

Next, we examined whether the fluorescent signal in labeled extracellular tachyzoites originated from the intact NBD-lipids and was not simply due to the degradation of probes in the extracellular or intracellular milieu. Indeed, thin layer chromatography (TLC) of total lipids extracted from the labeled tachyzoites identified NBD-PtdCho and NBD-SM in related samples (Fig. 1*C*), whose low intensity correlated with the microscopy and flow cytometry data. In contrast, a strong band of NBD-PtdEtn was detected in the corresponding parasite sample. Equally, tachyzoites incubated with NBD-PtdSer displayed a distinct band for NBD-PtdSer beside NBD-PtdEtn. The fact that PtdSer decarboxylation can occur in the mitochondrion, in dense granules, and in the PV when intracellular (9, 40), NBD-PtdSer supplied in the environment is possibly imported first, transported to the site of decarboxylation, and then decarboxylated to make NBD-PtdEtn (see later discussion). In none of the two samples, however, NBD-PtdEtn was methylated to yield NBD-PtdCho, confirming the lack of a PtdEtn methyltransferase in tachyzoites (8). In addition to specified lipids in their intact form, we also detected fluorescent fatty acids, probably as a result of lipolytic degradation.

Formation of NBD-PtdEtn in extracellular tachyzoites incubated with NBD-PtdSer can be attributed to the presence of PtdSer decarboxylase (PSD) enzymes in the mitochondrion and dense granules, the latter of which is ultimately secreted into the parasitophorous vacuole (9, 40). We tested the notion by NBD-PtdSer labeling of parasites in the presence of hydroxylamine (Fig. S2*A*), which is known to inhibit PSD activity (41). As expected, TLC analysis revealed that the conversion of NBD-PtdSer to NBD-PtdEtn was nearly ablated by the addition of the drug. The ratio of NBD-PtdEtn to NBD-PtdSer was about 60% to 40% in the untreated sample, whereas this proportion was changed to 15% *versus* 85% in the presence of hydroxylamine (Fig. S2*A*). To test the extent of decarboxylation occurring before or during NBD-PtdSer transport, we collected the secreted fraction (secretome), as described earlier (9, 40), and incubated it with the fluorescent probe (Fig. S2*B*). Indeed, consistent with our earlier work, we noticed the decarboxylation of PtdSer by *Tg*PSD1_pv_ discharged in the secretome; however, the degree of PtdEtn formation was much lower under the indicated labeling conditions when compared with the intact parasites, which harbor *Tg*PSD1_mt_ in the mitochondrion in addition to *Tg*PSD1_pv_ in dense granules (9, 40). These results, taken together, suggest that tachyzoites are competent in salvaging PtdSer that is subsequently metabolized to PtdEtn.

### Import of PtdEtn and PtdSer by tachyzoites is energy and protein dependent

**Figure 2:**
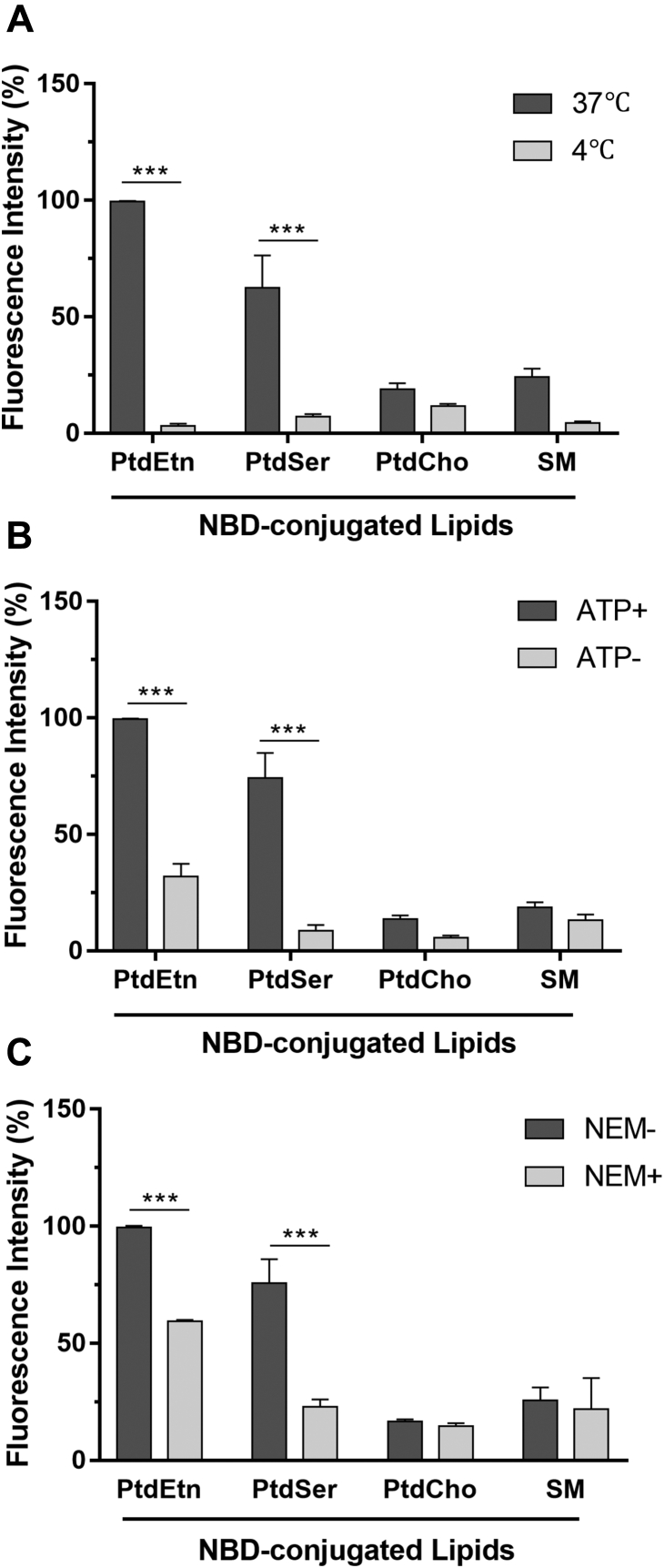
**Lipid internalization in *T. gondii* is a temperature-, ATP-, and protein-driven process.** Tachyzoites were incubated with NBD-lipids (30 min) followed by flow cytometric analysis. *A*, effect of temperature on the import of NBD-lipids by extracellular tachyzoites. *B*, ATP-dependence of lipid uptake. For the ATP content in corresponding samples (run alongside) refer to Figure S2*C*. *C*, influence of N-ethylmaleimide (NEM) treatment on the import of NBD-lipids. Data shown here are normalized to NBD-PtdEtn samples (set as 100%) at 37 °C (*A*), ATP-replete (*B*), or NEM-treatment (*C*) conditions. *A*–*C*, illustrate means ± SE from three assays (∗∗∗*p* ≤ 0.001).

To examine whether the uptake of lipid probes by extracellular parasites is an active process driven by ATP and proteins, we set up experiments under varying conditions (Fig. 2). In the first assay, we compared internalization of NBD-conjugated PtdCho, PtdEtn, PtdSer, and SM either at physiological (37 °C) or at low (4 °C) temperature by quantifying the fluorescence of each probe *via* flow cytometry (Fig. 2*A*). Parasites labeled with NBD-PtdCho or NBD-SM displayed a low (background) fluorescence at 37 °C that was further quenched at 4 °C. By contrast, cells incubated with NBD-PtdEtn and NBD-PtdSer displayed a notably higher signal and incurred a nearly complete loss of lipid salvage at low temperature. In a similar experiment when tachyzoites were incubated at 37 °C in ATP-replete and depleted media (Fig. S2*C*), we scored a marked decline in internalized fluorescence of NBD-PtdEtn and NBD-PtdSer but not of NBD-PtdCho and NBD-SM in energy-depleted tachyzoites (Fig. 2*B*). Finally, we incubated parasites with N-ethylmaleimide (NEM), a sulfydryl reagent that covalently alters the cysteine residues and thereby blocks disulfide cross-link in proteins (42), prior to the import assays (Fig. 2*C*). Once again, we recorded a specific decline in the uptake of NBD-conjugated PtdEtn and PtdSer of drug-treated tachyzoites. In conclusion, these experiments indicate the presence of ATP-dependent PtdSer and PtdEtn transporter proteins in the plasma membrane of tachyzoites.

### *Toxoplasma* genome encodes five potential P4-ATPases

**Figure 3:**
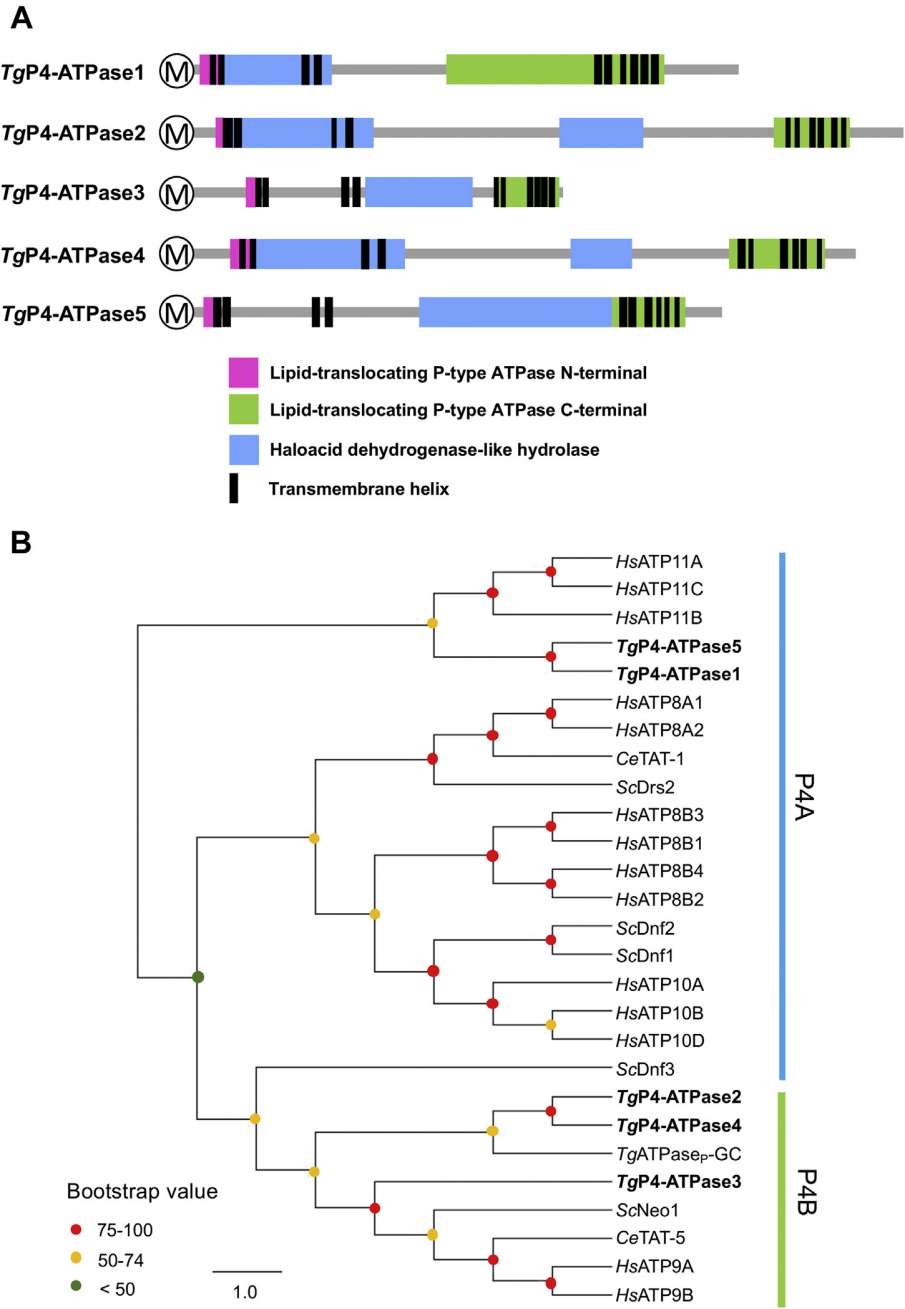
***T. gondii* encodes five potential phospholipid flippases.***A*, primary structure of *Tg*P4-ATPase1-5 encoded by the parasite. Functional domains were predicted by NCBI conserved domain search, PFAM v32.0 and SMART programs, and the number and location of transmembrane helices are based on the TMHMM, TMpred, and Phobius suites. *B*, a single parsimonious cladogram depicting the relationship of *Tg*P4-ATPase1-5 with homologs from *H. sapiens*, *S. cerevisiae*, and *C. elegans*. *Colored bars* indicate a classification of P4-ATPases, and dots on branch nodes show the bootstrap values. The bar scale signifies the amino acid substitutions per site. NCBI Accession: *Tg*P4-ATPase1, MT268297; *Tg*P4-ATPase2, MT268298; *Tg*P4-ATPase3, MT268299; *Tg*P4-ATPase4, MT268300; *Tg*P4-ATPase5, MT268301; *Hs*ATP8A1, Q9Y2Q0; *Hs*ATP8A2, Q9NTI2; *Hs*ATP8B1, O43520; *Hs*ATP8B2, P98198; *Hs*ATP8B3, O60423; *Hs*ATP8B4, Q8TF62; *Hs*ATP9A, O75110; *Hs*ATP9B, O43861; *Hs*ATP10A, O60312; *Hs*ATP10B, O94823; *Hs*ATP10D, Q9P241; *Hs*ATP11A, P98196; *Hs*ATP11B, Q9Y2G3; *Hs*ATP11C, Q8NB49; *Sc*Drs2, P39524; *Sc*Neo1, P40527; *Sc*Dnf1, P32660; *Sc*Dnf2, Q12675; *Sc*Dnf3, Q12674; *Ce*TAT-1, Q9U280; *Ce*TAT-5, G5EBH1.

In continuation of the above-mentioned findings, we searched the genome database of *T. gondii* for P4-ATPase proteins, which are known to transport phospholipids in other organisms. Our BLAST search using the well-studied lipid-translocating P4-ATPases (*Hs*ATP8A1 and *Sc*Drs2) identified five putative lipid transporters of variable length (1261–2427 aa, Table 1). These proteins, termed *Tg*P4-ATPase1-5 herein, comprise P4-ATPase domains at their N and C termini, flanking a haloacid dehydrogenase-like hydrolase domain (P-type ATPase superfamily) (Fig. 3*A*). All five *Tg*P4-ATPases harbor ten potential transmembrane (TM) helices (Fig. S3), as described in their human and yeast homologs. The first four TMs occur at the N terminal of proteins, whereas the subsequent six TMs are systematically positioned within the C-terminal P4-ATPase domain (Fig. 3*A*). Besides, the N and C termini of all flippases are predicted to face the cytoplasmic leaflet (Fig. S3). In a parsimonious cladogram, *Tg*P4-ATPase1 and *Tg*P4-ATPase5 paired with each other and branched off the *Hs*ATP11 family (P4A class, Fig. 3*B*), while the *Tg*P4-ATPase2-4 proteins formed own clades diverging off the mainstream P4B-type flippases (*Hs*ATP9A, *Hs*ATP9B, *Ce*TAT-5, *Sc*Neo1).

**Table 1:** Major features of *Toxoplasma* P4-ATPases identified in this study

Protein name	NCBI accession (ToxoDB ID)	Amino acids (size in kDa^a^)	Growth score^b^	Transcript (FPKM)^c^	Special features^d^	Partner protein (ToxoDB ID)^e^	Protein expression in tachyzoites^f^
P4-ATPase1	MT268297 (TGGT1_247690)	1860 (205)	−1.50	42.00	GYAFS (R domain)	---	Apical plasmalemma
P4-ATPase2	MT268298 (TGGT1_245510)	2427 (266)	−3.76	26.36	GFAFA (R domain)	---	Plasma membrane & endo/cytomembranes
P4-ATPase3	MT268299 (TGGT1_224190)	1261 (140)	−5.24	21.43	---	*Tg*Lem3^g^ (TGGT1_239540)	Golgi network
P4-ATPase4	MT268300 (TGGT1_257720)	2261 (250)	−0.38	00.05	Induced during chronic phase^h^	---	Not expressed in tachyzoites
P4-ATPase5	MT268301 (TGGT1_216380)	1803 (198)	−0.43	11.53	---	---	Plasma membrane & endo/cytomembranes

a Predicted molecular weight without any epitope.

b Based on genome-wide CRISPR screen in tachyzoites (58).

c Transcript level of fragments per kilobase of exon model per million mapped reads (57).

d Special features of shown P4-type proteins (see Discussion).

e There are four predicted Cdc50/Lem3-like proteins encoded by parasite genome (see Discussion).

f Deduced by 3′-insertional epitope tagging of *Tg*P4-ATPase1-5 genes.

g Based on localization of *Tg*Lem3 and *Tg*P4-ATPase3 in the Golgi network.

h As reported by Pittman *et al*. (66).

### *Tg*P4-ATPase1-5 harbor all functional motifs needed for lipid flipping

P4-ATPase protein (α subunit) contains a membrane (M) domain with 10 TM helices and a cytosolic region comprising the phosphorylation (P), nucleotide-binding (N), and actuator (A) domains (43, 44, 45). All these domains were found conserved with similar topological orientation in *Tg*P4-ATPase1-5 (Fig. S3). The A domain spans TM1-TM3, whereas the P and N domains reside between TM4 and TM5 in all five proteins. To discern their functionality, we mapped the signature motifs and residues by alignment with *Hs*ATP8A1 and *Sc*Drs2 (Fig. S4). The DKTGT motif containing an aspartate that undergoes the phosphorylation–dephosphorylation cycle in the P domain (a feature of all P-type ATPases) is strictly conserved in the parasite proteins. Other common motifs in the P domain (TGDx and GDGxND) that bind Mg^2+^ and link the ATP-binding region to the transmembrane segments are also present in *Tg*P4-ATPases. Evenly, the DGET-like motif in the A domain enabling dephosphorylation of the aspartate residue in the DKTGT motif and thereby facilitating the lipid flipping is broadly conserved in parasite flippases. Likewise, the PISL-like motif located explicitly in the TM4 helix (M domain) and harboring a proline residue involved in the protein folding and lipid flipping events (46, 47) occurs in all *Tg*P4-ATPases (Figs. S3 and S4). Finally, the K10.13039/100011271GA signature in the N domain, where lysine supports the docking of ATP for the DKTGT phosphorylation (48, 49), could also be identified in the *Toxoplasma* proteins.

Our further analysis disclosed that, of the two glutamine (QQ) residues located in the TM1 entry gate position that is needed for the selection of PtdSer in *Hs*ATP8A1 (25) and *Sc*Drs2 (50), only the first one is preserved in *Tg*P4-ATPases as is the case with *Sc*Neo1 (Fig. S4). Other determinants of substrate specificity, flippase activity, and exit gate position in TM2 and TM4 (47, 50, 51) also differ among all shown P4-ATPases (boxed residues, Fig. S4). Not least, as known in *Sc*Drs2p-Cdc50p and *Hs*ATP8A1-Cdc50a heterodimers, the entire complex normally exists in an autoinhibited state achieved by wrapping of the C-terminal regulatory (R) sequence around the A, P, and N domains (24, 26). The GYAFS motif in the R domain that underlies autoinhibitory regulation is fully conserved in *Tg*P4-ATPase1. A minor variation (GFAFA) was also seen in *Tg*P4-ATPase2, but other flippases are devoid of such a motif (Fig. S3-S4). Integrating all the above-mentioned findings, we can envisage *Tg*P4-ATPase1-5 as being functional lipid translocases, of which at least *Tg*P4-ATPase1-2 appear to be regulated by an activation factor (see Discussion).

### Tachyzoites express four *Tg*P4-ATPases localized in distinct organelles

**Figure 4:**
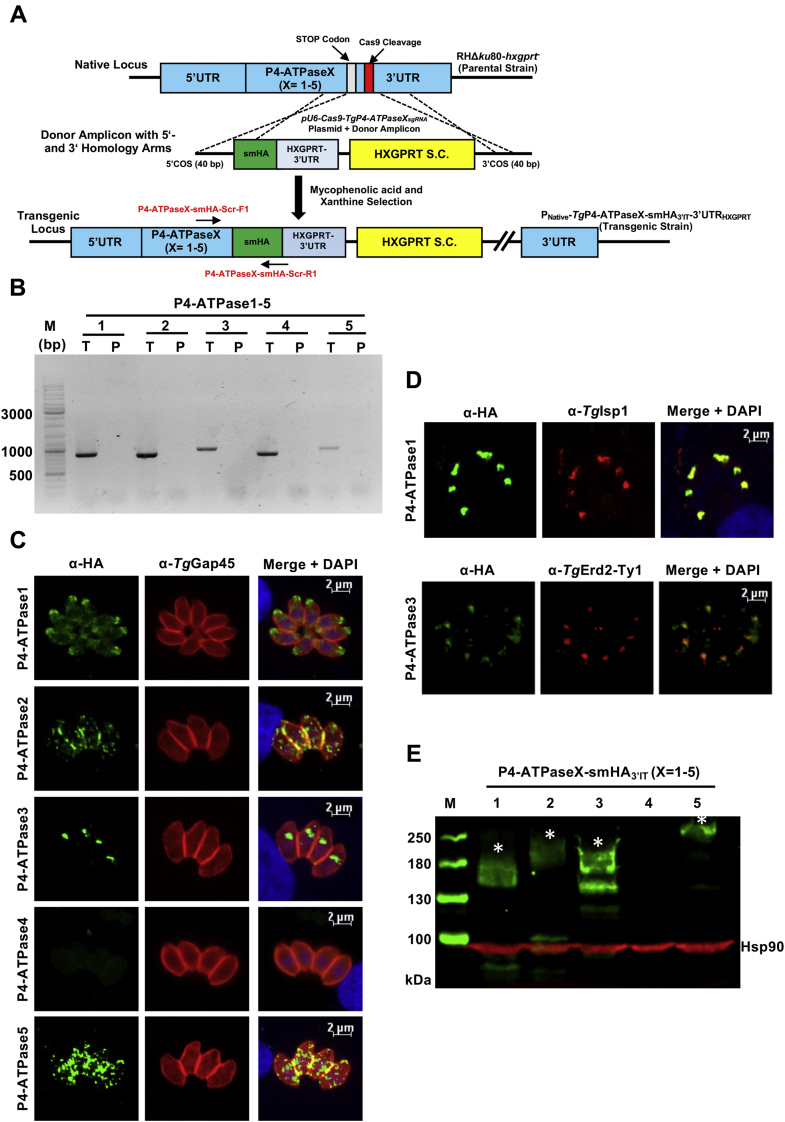
**P4-ATPases expressed in tachyzoite exhibit different subcellular distribution.***A*, 3′-Insertional tagging (3′IT) of *Tg*P4-ATPase1-5 genes with 10xHA epitope *a.k.a.* spaghetti monster HA (smHA). Individual constructs encoding for Cas9 and gene-specific *sg*RNA were cotransfected with PCR amplicons comprising smHA and HXGPRT selection cassette (S.C.) into the RHΔ*ku80*-*hxgprt*^−^ strain. Transgenic parasites were drug-selected and cloned by limiting dilution for further assays. *B*, genomic PCR screening to show the occurrence of homologous recombination in clonal transgenic strains expressing smHA-tagged *Tg*P4-ATPase1-5. Location of the screening primer pair is denoted in (*A*). Genomic DNA of the parental RHΔ*ku80*-*hxgprt*^*−*^ strain was included as a negative control. The letters “T” and “P” denote transgenic and parental strains, respectively. *C*, immunofluorescence of intracellular parasites expressing either of the five *Tg*P4-ATPase-smHA_3′IT_. Tachyzoites replicating in human foreskin fibroblast cells were fixed 24 h post infection and immunostained with α-HA and α-*Tg*Gap45 antibodies. *D*, costaining of *Tg*P4-ATPase1 and *Tg*P4-ATPase3 with selected organelle markers. Parasites expressing *Tg*P4-ATPase1-smHA_3′IT_ were stained with α-HA and α-*Tg*Isp1 antibodies. To colocalize *Tg*P4-ATPase3-HA_3′IT_, parasites were transfected with a plasmid expressing *Tg*Erd2-Ty1 and costained using α-HA and α-Ty1 antibodies. In (*C* and *D*), DAPI represented by *blue* in merged pictures was used for nuclei staining. *E*, immunoblot of parasites encoding *Tg*P4-ATPase-smHA_3′IT_ proteins. The protein extract prepared from extracellular tachyzoites (10^7^) were resolved by 6% SDS-PAGE, followed by immunoblotting using α-HA and α-*Tg*Hsp90 (loading control) antibodies. DAPI, 4′,6-diamidino-2-phenylindole.

To examine the expression and subcellular location of *Tg*P4-ATPases in tachyzoites of *T. gondii*, we first performed single crossover-mediated genomic tagging with a C-terminal hemagglutinin (HA) tag (Fig. S5*A*). Of the five proteins, *Tg*P4-ATPase1-3 could be successfully tagged, as confirmed by PCR screening using recombination-specific primers (Fig. S5*B*). *Tg*P4-ATPase1-HA_3′IT_-3′UTR_floxed_ strain, for instance, yielded an ≈2.5-kb band. Similarly, *Tg*P4-ATPase2-HA_3′IT_-3′UTR_floxed_ and *Tg*P4-ATPase3-HA_3′IT_-3′UTR_floxed_ strains revealed amplicons of ≈2.5 and ≈2.6 kb, respectively. Immunostaining showed that *Tg*P4-ATPase1-HA_3′IT_ was distributed mainly at the anterior pole of tachyzoites, and *Tg*P4-ATPase2-HA_3′IT_ was located likely in the plasma membrane, as judged by partial staining with a marker of the inner membrane complex (IMC), *Tg*Gap45 (52) (Fig. S5*C*). *Tg*P4-ATPase3-HA_3′IT_ on the other hand was detected above the nucleus (in the Golgi network, see later discussion). *Tg*P4-ATPase2 exhibited a weak staining, whereas *Tg*P4-ATPase4 and *Tg*P4-ATPase5 could not be tagged or detected. Hence, a high-affinity spaghetti monster HA tag (53) was inserted into the 3′-end of all genes, which allowed detection of all five P4-ATPases (Fig. 4*A*). The recombination-specific PCR and sequencing verified the epitope tagging (Fig. 4*B*). In plaque assays, none of the strains exhibited a growth defect, ruling out a detrimental effect of the smHA tag on the flippase function in tachyzoite cultures (Fig. S6).

Immunofluorescence imaging disclosed that *Tg*P4-ATPase1-smHA_3′IT_ is located at the parasite apex along with faint expression in the perinuclear region (Fig. 4*C*). Its apical distribution was confirmed by costaining with a marker of the apical cap, IMC subcompartment protein 1 (*Tg*Isp1) (54) (Fig. 4*D*). *Tg*P4-ATPase2-smHA_3′IT_ was expressed in the plasma membrane alongside the endo/cytomembranes (Fig. 4*C*). *Tg*P4-ATPase3-smHA_3′IT_ was shown to be in the Golgi apparatus by staining with Ty1-tagged *Tg*Erd2 (55) (Fig. 4, *C* and *D*). Its potential β subunit *Tg*Lem3 was also present in the Golgi network (Fig. S7, *A* and *B*). *Tg*P4-ATPase4-smHA_3′IT_ signal was not visible in tachyzoites in spite of a successful tagging, whereas *Tg*P4-ATPase5-smHA_3′IT_ was localized in the plasmalemma and endo/cytomembranes (punctate expression). Of note, parasites encoding *Tg*P4-ATPase2-smHA_3′IT_ and *Tg*P4-ATPase5-smHA_3′IT_ also exhibited a strong signal in the peripheral region at the intersection of progeny (merged yellow signal, Fig. 4*C*). Besides, immunoblotting of *Tg*P4-ATPase1-3 and *Tg*P4-ATPase5 revealed clear signals, with the largest protein bands corresponding to 180, 200, 190, and 270 kDa, respectively (see *asterisks*, Fig. 4*E*). The anomalous electrophoretic mobility compared with their predicted size (Table 1) and potential degradation products (see smaller bands in Fig. 4*E*) are possibly due to the presence of multiple transmembrane helices, as explained elsewhere (56). Once again, the expression of *Tg*P4-ATPase4 was not detectable in immunoblot either, which resonates with its nearly absent transcript level in tachyzoites of *T. gondii* (57).

### *Tg*P4-ATPase1-2 and 5 reside in the plasma membrane with the C terminus facing the cytosol

**Figure 5:**
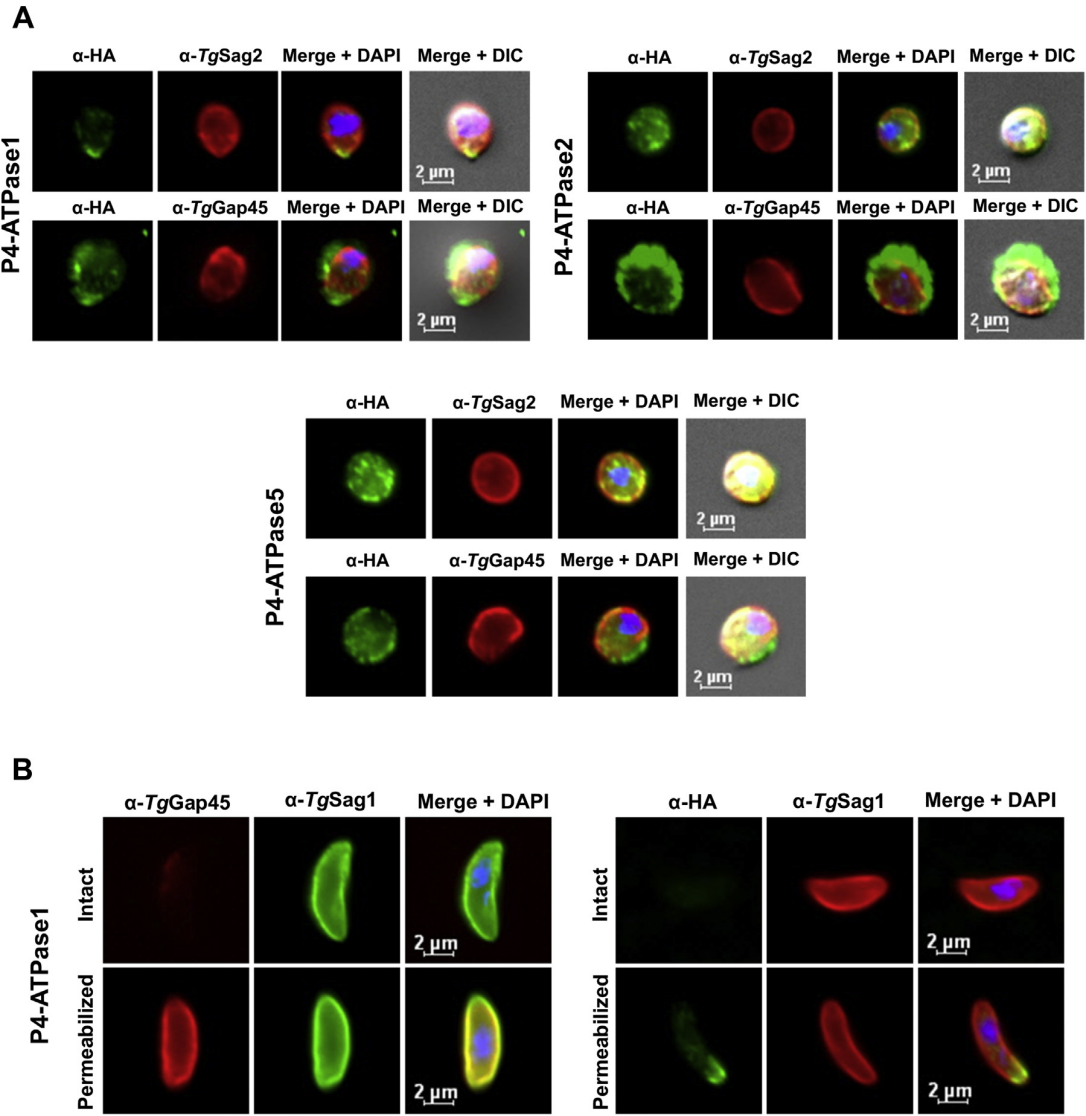
**P4-ATPase1-2 and 5 localize in the plasma membrane**. *A*, subcellular distribution of chosen *Tg*P4-ATPase-smHA_3IT_ proteins after the drug-induced splitting of the plasmalemma and inner membrane complex (IMC). Parasites were incubated with α-toxin (20 nM, 3 h) before staining for the HA tag in combination with the antibodies recognizing IMC (*Tg*Gap45) or plasma membrane (*Tg*Sag2). *B*, experimental testing of the C-terminal orientation in *Tg*P4-ATPase1-smHA_3IT_ protein. Tachyzoites were immunostained after treating with detergent-free PBS (“Intact”) or detergent-PBS (“Permeabilized”). The parasite and host-cell nuclei were visualized by DAPI (*blue*) in *A-B*. DAPI, 4′,6-diamidino-2-phenylindole.

The apical presence of *Tg*P4-ATPase1 indicated plasmalemma as a probable site of its expression. *Tg*P4-ATPase2 and 5 also showed partly peripheral staining while localizing with *Tg*Gap45 (Fig. 4*C*). To discern their exact location, we treated parasites with α-toxin to split the IMC from the plasma membrane (PM) and thereby distinguish the distribution of *Tg*P4-ATPase1-2/5 in the two entities. Subsequent immunostaining of the IMC (*Tg*Gap45) and PM (*Tg*Sag2) marker proteins suggested the presence of *Tg*P4-ATPase1-2/5 in the plasma membrane (Fig. 5*A*). In further work, prompted by a well-defined *regulatory* (R) domain at the C terminus of *Tg*P4-ATPase1 and its unique location, we experimentally tested its membrane topology. In this regard, we stained parasites before and after detergent-induced membrane permeabilization (Fig. 5*B*). As expected, the *Tg*Gap45 signal was seen only after permeabilization, whereas *Tg*Sag2 was visible both in the permeabilized as well as in the intact cells. The HA staining of *Tg*P4-ATPase1 on the other hand was detected only after permeabilization, confirming that the C terminus of P4-ATPase1 indeed faces the cytoplasmic side of the plasma membrane (Fig. 5*B*), as predicted by our *in silico* analysis (Fig. S3).

### *Tg*P4-ATPase1-3 proteins are required for asexual reproduction of tachyzoites

**Figure 6:**
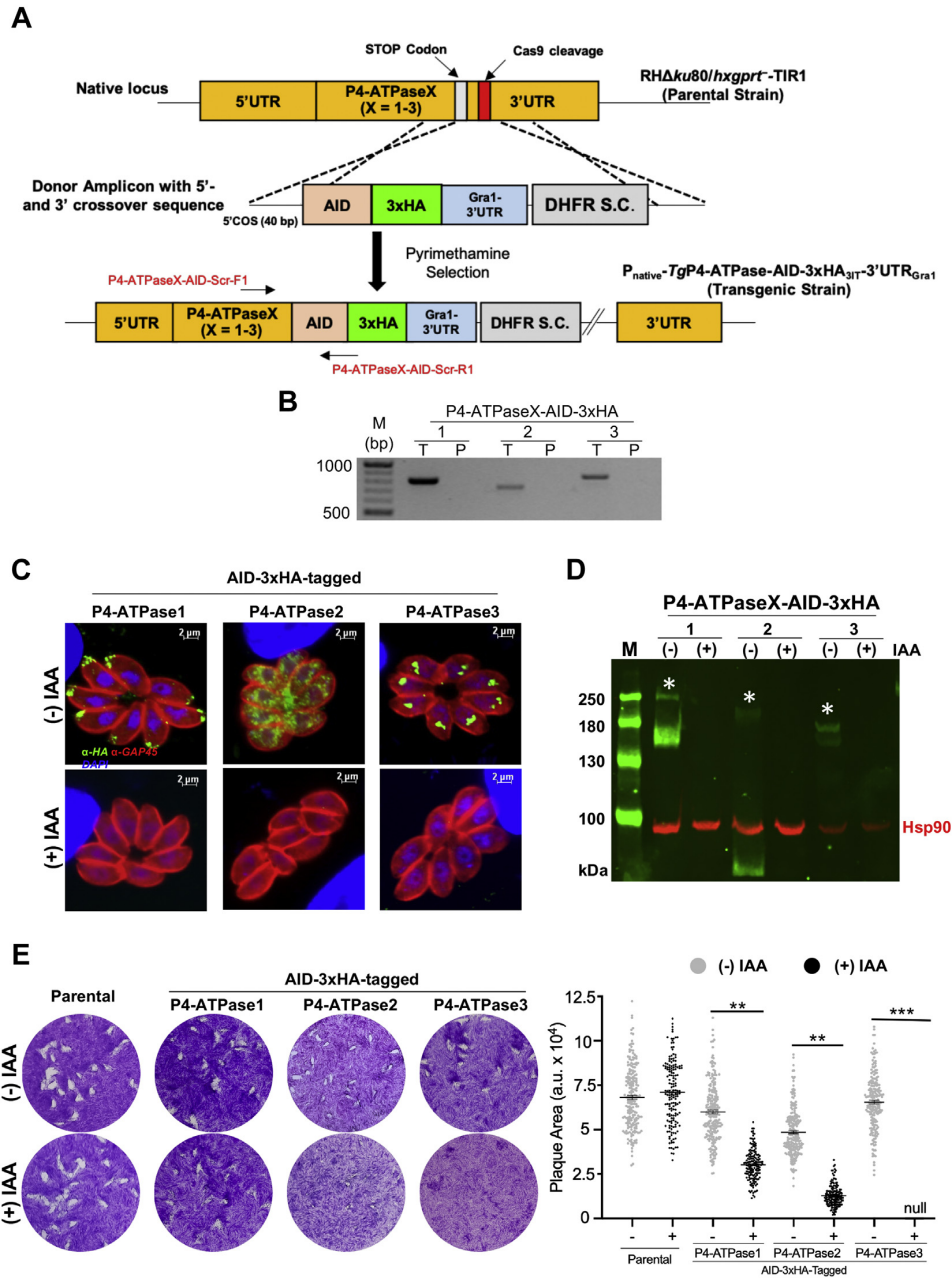
**Knockdown of *Tg*P4-ATPase1-3 is detrimental to the tachyzoite growth**. *A*, scheme for CRISPR-Cas9–assisted 3′-insertional tagging of *Tg*P4-ATPase1-3 with an auxin-inducible degron (AID) and 3xHA. *pU6-Cas9-TgP4-ATPaseX*_*sgRNA*_ plasmids (encoding Cas9 and gene-specific *sg*RNA) were cotransfected with matching donor amplicons (AID-3xHA-3′UTR_Gra1_-DHFR-TS) flanked by a 40-bp crossover sequence (COS) into a parental strain (RHΔ*ku80*-*hxgprt*^*−*^-TIR1), and drug-selected for the DHFR-TS selection cassette (S.C.) using pyrimethamine. Transgenic tachyzoites (P_native_-*Tg*P4-ATPaseX-AID-3xHA_3′IT_-3′UTR_Gra1_, X = 1–3) expressed AID-3xHA-tagged *Tg*P4-ATPases, which could be conditionally regulated by auxin. *B*, verification of the Cas9-assisted AID-3xHA tagging of genes in mutants by PCR screening using specific primer pairs (see *A*). The letters “T” and “P” denote transgenic and parental strains, respectively. *C*, auxin-dependent expression of AID-3xHA-tagged *Tg*P4-ATPase proteins. Parasites were cultured with or without 500 μM IAA for 24 h and then stained with α-HA (*green*) and α-*Tg*Gap45 (*red*) antibodies and DAPI (*blue*). *D*, immunoblots of transgenic strains before and after auxin treatment. Equivalent amounts of protein for each sample were resolved on 6% SDS-PAGE, followed by blotting and immunostaining of HA and *Tg*Hsp90 (loading control). The *asterisks* denote the size of 250, 200, and 170 kDa for epitope-tagged forms of *Tg*P4-ATPase1-3, respectively. Note the difference in the size of *Tg*P4-ATPase1-AID-3xHA in comparison with the smHA-tagged form, which did not reveal a band of 250 kDa (Fig. 4*E*). *E*, plaques formed by the mutant and parental strains in the absence or presence of IAA. Crystal violet–stained images reveal plaques formed by successive lytic cycles of the stated strains. The plaque area, scored by ImageJ, is depicted in arbitrary units (a. u.). About 150 to 200 plaques of each strain were evaluated (n = 3 assays; means ± SE; ∗∗*p* ≤ 0.01, ∗∗∗*p* ≤ 0.001). DAPI, 4′,6-diamidino-2-phenylindole; DHFR-TS, dihydrofolate reductase–thymidylate synthase.

Given the explicit subcellular expression and negative phenotypic score of *Tg*P4-ATPase1-3 proteins (Table 1), we examined their physiological relevance in tachyzoites. We first endeavored to ablate the relevant gene loci by homologous recombination but could not obtain the viable mutants, implying the essential function of the three flippases in agreement with the genome-wide knockout screening (58). Hence, we deployed an auxin-inducible degron (AID), which upon 3′-genomic tagging results in a rapid conditional knockdown of the conjugated protein by proteasomal degradation in tachyzoite cultures supplemented with indole-3-acetic acid (IAA) (59). Cotransfection of a matching parental strain (RHΔ*ku80*-*hxgprt*^*−*^*-*TIR1) with individual AID-3xHA tagging amplicons along with conforming *sg*RNA-expression constructs resulted in the engineering of *Tg*P4-ATPaseX-AID-3xHA mutants (X = 1–3, Fig. 6*A*), as endorsed by genomic screening (Fig. 6*B*). Unlike the parental strain (control), *Tg*P4-ATPaseX-AID-3xHA mutants displayed PCR bands of the expected amplicon size (≈700–800 bp), which were further corroborated by DNA sequencing.

Immunofluorescence staining of AID-3xHA-tagged *Tg*P4-ATPase1-3 proteins once more confirmed their subcellular expression (Fig. 6*C*), as observed in 1xHA and smHA-tagged strains (Figs. S5*C* and 4*C*). More importantly, the fluorescent signal was not detectable upon treatment with IAA. Immunoblot of the clonal knockdown mutants showed the protein bands, as anticipated, all of which became absent following the auxin supplementation (Fig. 6*D*). Next, we tested whether repression of individual *Tg*P4-ATPase1-3 proteins would translate into a growth defect in plaque assays (Fig. 6*E*). As envisaged, the parental strain was unaffected by IAA exposure. In contrast, *Tg*P4-ATPase1 and *Tg*P4-ATPase2 mutants unveiled *ca.* 50% and 80% reduction in their plaque size, respectively, during the *off state* (+IAA) when compared with the parental control, whereas the growth of *Tg*P4-ATPase3 was fully ablated. Indeed, the former two mutants showed a modest impairment even in the absence of auxin, which can be attributed to genomic tagging with AID-3xHA and insertion of a foreign 3′UTR. Markedly, the spatial arrangement of the progeny tachyzoites, which usually form a rosette within a vacuole, was disrupted upon downregulation of *Tg*P4-ATPase2 (Fig. 6*C*). In summary, all these data reveal a physiological requirement of *Tg*P4-ATPase1-3 proteins for the lytic cycle of tachyzoites.

## Discussion

**Figure 7:**
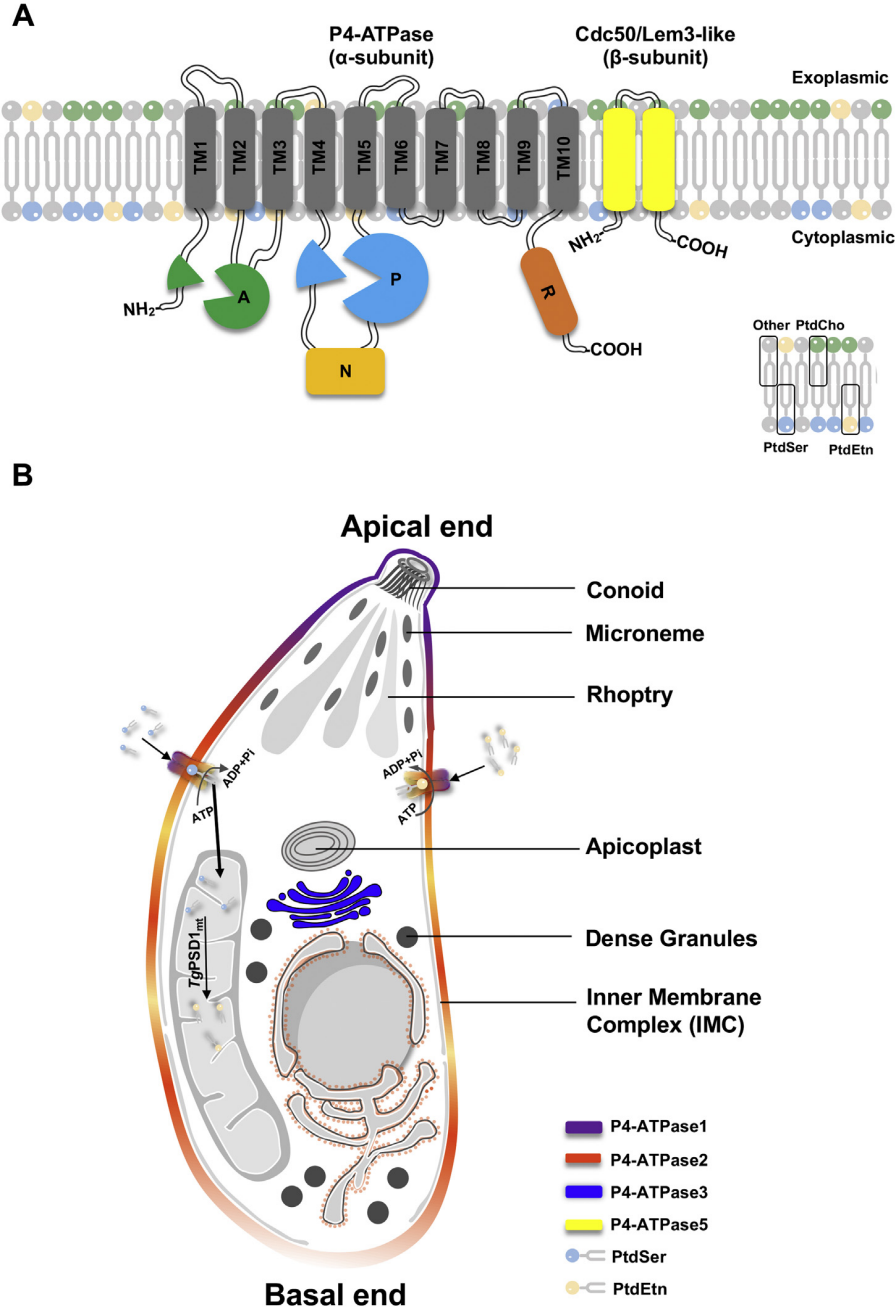
***Tg*P4-ATPases expressed in tachyzoites of *T. gondii*.***A*, predicted topologies of *Tg*P4-ATPase proteins. The M (membrane), A (actuator), P (phosphorylation), and N (nucleotide-binding) domains are present in all five parasite flippases, but the domain R (regulatory) occurs only in *Tg*P4-ATPase1-2. *B*, Subcellular distribution of *Tg*P4-ATPase1-3 and 5 proteins in the tachyzoite stage. *Tg*P4-ATPase1 localizes in the apical plasma membrane, whereas *Tg*P4-ATPase3 and its noncatalytic β subunit (*Tg*Lem3) reside in the Golgi network. *Tg*P4-ATPase2 and *Tg*P4-ATPase5 are distributed in the plasmalemma and endo/cytomembranes. *Tg*P4-ATPase4 is not expressed in the tachyzoite stage. Import of PtdSer and PtdEtn is mediated by surface-resident flippases in an ATP-dependent manner. According to the shown model, PtdSer salvaged from the external milieu can be decarboxylated by *Tg*PSD1_mt_ to produce PtdEtn, likely in the mitochondrion. Note that decarboxylation reaction can also happen, at least in part, in the parasite milieu by secreted *Tg*PSD1_pv_. *PSD*, phosphatidylserine decarboxylase.

This work characterized the aminoglycerophospholipid flipping in *T. gondii* (Table 1, Fig. 7). Its acutely infectious tachyzoite stage encodes at least four P4-ATPases of which three are localized in the plasma membrane. Accordingly, PtdEtn and PtdSer supplied in the milieu traffic to the parasite interior probably *via* any of the surface-resident flippases described here. The latter lipid is eventually decarboxylated to produce PtdEtn, highlighting the ability of *T. gondii* to utilize external phospholipids for its membrane biogenesis. Previous work in yeast has demonstrated that exogenous lyso-PtdEtn and lyso-PtdCho salvaged by PM-localized *Sc*Dnf1 and *Sc*Dnf2 in consort with *Sc*Lem3 can serve as the substrates for phospholipid synthesis (30, 31). Specifically, internalized lysophospholipids are acylated to yield PtdEtn and PtdCho, and their transport rates are sufficient to support the membrane biogenesis and growth of *S. cerevisiae*. Similarly, *T. gondii* tachyzoites satisfy their lipid requirements by *de novo* synthesis as well as salvage from the environment (5, 6, 7, 8, 9, 10, 11, 12, 13, 14). Soon after the invasion, the PV membrane becomes intimately associated with the host mitochondria and endoplasmic reticulum (60), which are two major sites of phospholipid biosynthesis in mammalian cells (61). We have shown a functional (coccidian-specific) PtdSer decarboxylase located in the PV that produces PtdEtn using PtdSer as the substrate (9, 40). Import of PtdSer and PtdEtn by means of surface P4-ATPases and their ensuing metabolism are therefore fairly conceivable in tachyzoites. Our work suggests that *T. gondii* may exploit selected flippases to salvage host-derived lipids for its asexual reproduction.

We determined that *Tg*P4-ATPase1 and *Tg*P4-ATPase2 are critical, whereas *Tg*P4-ATPase3 is essential for the lytic cycle. Several studies, primarily in yeast and mammalian cells, have shown that flippases in the plasmalemma and in other organelles govern assorted subcellular events, and perturbed lipid distribution in membranes can impact apoptosis, cytokinesis, membrane fusion, vesicular traffic, and endocytosis (43, 45). In our context, transbilayer asymmetry of PtdEtn, which induces the membrane curvature needed for vesicle formation during secretory traffic (62), may be relevant during exocytosis of micronemes and rhoptries at the apical pole of tachyzoites (63). This process can potentially be facilitated by *Tg*P4-ATPase1 at the parasite apex. Our finding of disorganized tachyzoite rosettes in the *Tg*P4-ATPase2 mutant resonates with *Tg*MORN1 and *Tg*UNC mutants (64, 65), signifying that *Tg*P4-ATPase2 could underlie the formation or maintenance of the basal complex in *T. gondii*. *Tg*P4-ATPase2 shares its subcellular expression with *Tg*P4-ATPase5, implying a functional redundancy, which remains to be examined by combinatorial mutagenesis and lipid uptake assays. Expression of *Tg*P4-ATPase3 (and *Tg*Lem3) in the Golgi complex on the other hand indicates an engagement in the organelle-associated trafficking. Further work is required to ascertain the substrate specificity of these flippases in an appropriate heterologous model, *e.g.*, *S. cerevisiae*. Last but not least, *Tg*P4-ATPase4 is not transcribed in the tachyzoite stage but induced > fivefold during chronic infection of the mouse brain (66), indicating its importance in the quiescent bradyzoite stage that merits investigation in the cyst-forming strains of *T. gondii*.

Our search of other apicomplexan parasite genomes revealed the presence of three to four putative flippases in *Plasmodium falciparum*, *Eimeria tenella*, and *Cryptosporidium parvum*, signifying their conserved roles across the phylum. In particular, the signature motifs needed for the lipid flipping function are present in all indicated apicomplexan homologs of *Tg*P4-ATPase1 (Fig. S8). In harmony, they harbor a *regulatory* GYAFS-like motif akin to *Sc*Drs2p and *Hs*ATP8A1 (24, 25, 26). The latter proteins, existing usually in their autoinhibited states, are activated by phosphatidylinositol-4-phosphate (24, 25, 26, 67, 68), a key signaling mediator produced during IP_3_-regulated discharge of calcium into the cytosol and ensuing Ca^2+^-dependent microneme exocytosis in *T. gondii* (69). In this context, a P4-ATPase-conjugated guanylate cyclase (ATPase_p_-GC or GC), also located in the apical plasma membrane, was recently reported to regulate the egress and invasion events that depend on exocytosis of micronemes in tachyzoites (70, 71, 72, 73). Albeit the lipid specificity of *Tg*ATPase_p_-GC and *Tg*P4-ATPase1 is still a mystery, the latter clusters with *Hs*ATP11 family flippases, which are proven to translocate PtdSer and PtdEtn (74, 75, 76), two lipids that we show here as readily internalized by tachyzoites. Based on our work and the above-mentioned findings in analogous flippases, we propose a functional engagement of *Tg*P4-ATPase1 during microneme exocytosis and associated events in *T. gondii*.

Our work also identified four Lem3/Cdc50-like proteins in *T. gondii* (TGGT1_230820, TGGT1_232040, TGGT1_239540, TGGT1_247380), three of which form an apicomplexan-specific clade deviating off the yeast and human orthologs (Fig. S7*C*). One of them (TGGT1_230820, *Tg*Cdc50.1) was reported to localize at the apical end and couple with GC *a.k.a. Tg*ATPase_p_-GC (73). *Tg*P4-ATPase1, located apically, may also functionally associate with *Tg*Cdc50.1. Equally, *Tg*Lem3 in the Golgi is expected to partner with *Tg*P4-ATPase3 based on the shared location. At the same time, the disparity in the number of α and β subunits implies that some parasite ATPases may not need a β subunit for their functionality, as known for *Hs*ATP9B (77) and *Sc*Neo1 (78). Other apicomplexans were also found to contain three to four Lem3/Cdc50 homologs (Fig. S7*C*). Quite notably, one *T. gondii* (TGGT1_247380), one *Eimeria*, and two *Cryptosporidium* orthologs branch off the large group of Lem3/Cdc50-like proteins, suggesting an evolutionary divergence and tailored requirement of lipid flipping in these pathogens. In *Leishmania donovani*, an intracellular parasite belonging to the protozoan phylum Kinetoplastida, antiparasitic effect of miltefosine obliges a phospholipid-translocating P4-ATPase and Lem3/Cdc50 homolog (79, 80). We found that miltefosine and edelfosine were broadly ineffective against the acute stage of *T. gondii*; however, the former drug was shown to be inhibitive against chronic toxoplasmosis (81). Further work is needed to test whether *Tg*P4-ATPases may offer an opportunity to target certain parasitic stages. The five P4-ATPases and lipid substrates described here provide a solid framework for future research on functional specificity and clinical intervention in *T. gondii*.

## Experimental procedures

### Biological resources and reagents

The RH strain of *T. gondii* and human foreskin fibroblasts (HFFs) were obtained from Carsten Lüder (Georg-August University, Göttingen). The RHΔ*ku80*-*hxgprt*^*−*^ strain, which lacks nonhomologous end joining repair and thus is more amicable to homologous recombination-mediated genetic manipulation (82, 83), was afforded by Vern Carruthers (University of Michigan). The RHΔ*ku80*-*hxgprt*^*−*^-TIR1 strain and the respective epitope-tagging vector for auxin-inducible conditional downregulation of essential proteins (59) were offered by David Sibley (Washington University in St Louis). The *pLIC-smHA-HXGPRT* vector for 3′-insertional tagging of genes with spaghetti monster HA (smHA/10xHA) was provided by Bang Shen (Huazhong Agricultural University). Other vectors encoding for *Tg*ERD2-Ty1 (*pTUB8-TgERD2-Ty1*) or expressing Cas9 and *sg*RNA (*pU6-Cas9*) were endowed by Boris Striepen (University of Pennsylvania) and David Sibley (Washington University in St Louis).

Antibodies recognizing *Tg*Hsp90, *Tg*Gap45, *Tg*Isp1, and *Tg*Sag2 proteins were provided by Sergio Angel (IIB-INTECH), Dominique Soldati-Favre (University of Geneva), Peter Bradley (University of California, Los Angles), and Honglin Jia (Harbin Veterinary Research Institute), respectively. Other antibodies recognizing the engineered epitopes (HA, Ty1) were obtained from Thermo Fisher Scientific and Sigma-Aldrich. Fluorophore-conjugated secondary antibodies (Alexa488, Alexa594) were purchased from Life Technologies. The culture medium and additives were procured from PAN Biotech. Lipids tagged with C6-NBD were acquired from Avanti Polar Lipids. DNA-modifying enzymes were obtained from New England Biolabs. Commercial kits for molecular cloning were delivered by Life Technologies and Analytik Jena. Oligonucleotides were synthesized by Thermo Fisher Scientific. Common chemicals were supplied by Sigma-Aldrich. All reagents were stored and utilized as per guidelines provided by the source.

### Host cell and parasite culture

Tachyzoites of *T. gondii* were propagated in confluent monolayers of HFF cells as reported (8). Host cells were grown to confluence in flasks, dishes, and plates as needed and harvested for routine culture by trypsin treatment. Uninfected and parasitized HFFs were cultured (5% CO_2_, 37 °C) in a humidified incubator in Dulbecco's Modified Eagle's Medium augmented with glucose (4.5 g/l), fetal bovine serum (10%), glutamine (2 mM), sodium pyruvate (1 mM), penicillin (100 U/mL), streptomycin (100 μg/mL), and minimum Eagle's minimum (MEM) nonessential amino acids (100 μM of each, serine, glycine, alanine, asparagine, aspartic acid, glutamate, proline). Unless stated otherwise, HFFs were infected with tachyzoites at a multiplicity of infection (MOI) of two, and for most assays the parasites were mechanically harvested from the late-stage cultures (40–44 h post infection).

### NBD-lipid uptake assay

To prepare extracellular tachyzoites, parasitized HFFs (MOI, 2; 40–44 h post infection) were scraped in the culture medium, squirted through 27-gauge syringe 3×, and then washed 2× with phosphate buffer saline (PBS; 1400*g*, 10 min, 4 °C). The parasite pellet was suspended in 950 μl of ice-cold labeling medium containing 20 mM Hepes, 140 mM KCl, 10 mM NaCl, 2.5 mM MgCl_2_, 0.1 μM CaCl_2_, 1 mM Na pyruvate, 1 mM ATP, 5 mM glucose, 1× MEM vitamins lacking choline, 1× MEM amino acids, and 1× serine-free nonessential amino acids. The last six ingredients were added fresh, and the pH of the labeling medium was adjusted to 7.4. The NBD-lipid probes (0.5–1 nmol/sample) were dried in conical-bottom glass tubes, suspended in ethanol (5 μl/sample), and then mixed with labeling medium (50 μl/sample) by extensive vortexing to prepare the substrate stock. Subsequently, the lipid stock was distributed into reaction tubes containing freshly released tachyzoites (10^7^) in 950 μl of labeling medium. The final reaction was incubated at 37 °C for 30 min in a shaking water bath.

The reactions were quenched by cooling on ice, and samples were washed 2× with 5% bovine serum albumin (BSA, w/v, 4 °C, 1400*g*, 10 min), followed by two further washing steps with labeling medium lacking the lipid probe. The cell pellets were eventually suspended in 500 μl medium and subjected to fluorescence microscopy, flow cytometry, and lipid analysis, as mentioned elsewhere. A negative control reaction without any lipid inclusion was also executed in parallel. To test the ATP dependence of the process, tachyzoites were preincubated for 30 min at 37 °C in a labeling medium that was either replete with 1 mM ATP, 5 mM glucose, and 1 mM Na-pyruvate or contained 2-deoxyglucose (5 mM), NaF (5 mM), and KCN (5 mM) before setting up the lipid uptake assay. To examine the protein dependence by modifying the cysteine residues, parasites were preincubated in medium with NEM (0.5 mM) for 15 min at 28 °C, washed once in medium without NEM, and then labeled with designated NBD-probes (37 °C, 30 min).

### Preparation of tachyzoite secretome

The secreted fraction of extracellular parasites was collected, as reported earlier (9, 40), to measure a contribution of *Tg*PSD1_pv_ activity during lipid uptake assays. Syringe-released parasites (40–44 h post infection) were pelleted (400 *g*, 15 min, 4 °C) and washed and suspended in 1.2 mL of lipid labeling medium (10^7^) supplemented with 1 mM ATP. Tachyzoites were allowed to discharge proteins for 30 min at 37 °C, pelleted (2000*g*, 10 min, 4 °C), and then 1.1 mL supernatant was carefully transferred into a new tube, whereas the sedimented parasites were stored on ice and later suspended in 900 μl medium to perform the lipid uptake studies. The secretome was centrifuged, and subsequently, a 1-mL fraction was collected, followed by yet-another centrifugation and transfer of 0.9 mL supernatant. Such serial centrifugation steps ensured a gradual removal of tachyzoites from the final secretome sample and thus minimized a lytic release of PSD activity. Nonetheless, a contribution of PSD activity due to centrifugal lysis of parasites (*i.e.*, not naturally secreted) during secretome preparation cannot be completely excluded.

### Imaging and flow cytometry of NBD-labeled parasites

Extracellular tachyzoites labeled with NBD-conjugated lipid probes (150–200 μl of the 1-mL reaction) were pipetted onto coverslips placed in 24-well plates. One microliter propidium iodide solution (0.1 mg/mL) was added to each sample to mark the dead cells. Plates were centrifuged (300*g*, 5 min) to enforce the parasite adhesion onto coverslips, which were then mounted in Fluoromount G/4′,6-diamidino-2-phenylindole for imaging. Slides were viewed under an AxioVision upright microscope equipped with ApoTome (Zeiss). For flow cytometry, each NBD lipid-labeled sample (100 μl) was mixed with 0.5 μl propidium iodide (0.1 mg/mL stock) and 350 μl of probe-free labeling medium. Twenty thousand cells were analyzed (FACScan, Becton Dickinson) without gating using two fluorescence channels (NBD, 530/30 nm; propidium iodide, 585/42 nm). Data were acquired with CellQuest software and processed using Cyflogic program (CyFlo Ltd). Main cell populations were gated based on the forward and side scattering values. Live cells were selected by propidium iodide exclusion. Green fluorescence (NBD) of living cells was plotted on a histogram, and the geometric mean of the fluorescence intensity was calculated. The background signal of negative control (no NBD-lipid) was subtracted from the mean fluorescence intensity of all lipid-labeled samples.

### Lipid extraction and thin-layer chromatography

Lipids were extracted according to Bligh and Dyer (84). Immediately after labeling assays, 0.5 mL of samples were mixed with 1.5 mL of chloroform/methanol (1:2, v/v), followed by the addition of 0.5 mL of 40 mM acetic acid and 0.5 mL of chloroform, each with rapid vortexing. Samples were allowed to stand still for about 15 min at room temperature to achieve the separation of liquid phases. About, 90% of the lower (chloroform) phase was transferred to a new glass tube using a Pasteur pipette and evaporated by a gentle nitrogen stream at 40 °C. The dry lipids were resuspended in 25 to 50 μl of chloroform/methanol (1:2, v/v) and loaded onto a TLC plate (Silica Gel 60H, Merck) together with commercial standards. Lipids were resolved in a solvent mixture containing chloroform, methanol, and water (65:25:4, v/v). The air-dried TLC plates were visualized by an imaging system (excitation, 488 nm; emission, 515 nm; FLA-3000, FujiFilm).

### Genomic tagging of *Tg*P4-ATPase1-5

The expression and subcellular location of *Tg*P4-ATPases were determined by 3′-insertional tagging (3′IT) of corresponding genes with a smHA epitope. Sequences of *Tg*P4-ATPase1-5 were obtained from the parasite database (ToxoDB) (85). The *pU6-Cas9-TgP4-ATPaseX*_*sgRNA*_ (X = 1–5) constructs expressing Cas9 and *sg*RNA targeting the 3′UTR of individual P4-ATPases were generated by Q5 site-directed mutagenesis (New England Biolabs). Gene-specific *sg*RNA-encoding oligonucleotides downstream of the stop codon were designed by a CRISPR guide RNA/DNA design tool (EuPaGDT). Subsequently, linear fragments of *pU6-Cas9-TgP4-ATPaseX*_*sgRNA*_ constructs were amplified using specific primers (*sg*RNA in the forward primer) and *pU6-Cas9* template and then cyclized by KLD enzyme as per the manufacturer's protocol. The PCR amplicons harboring smHA and hypoxanthine-xanthine-guanine phosphoribosyltransferase (HXGPRT) selection cassette, both flanked by 5′ and 3′-homology arms (40 bp) of individual flippases, were amplified using *pLIC-smHA-HXGPRT* plasmid template and Phanta Max Super-Fidelity DNA polymerase (Vazyme Biotech) (Table S1).

The conforming pairs of PCR amplicon (2 μg) and purified *sg*RNA-encoding construct (15 μg) were electroporated into tachyzoites suspended in filter-sterile Cytomix (120 mM KCl, 0.15 mM CaCl_2_, 10 mM K_2_HPO_4_/KH_2_PO_4_, 25 mM Hepes, 2 mM EGTA, and 5 mM MgCl_2_ with fresh 2 μM ATP and 5 μM glutathione, pH 7.4). Parasites were transfected by a cuvette-based Amaxa electroporation device (Lonza Group) using manufacturer's protocol (T-016 program, 1700 V, 50 Ω, 2 pulses of 176 μs at the interval of 100 ms, unipolar). Transgenic tachyzoites expressing HXGPRT were selected by mycophenolic acid (25 μg/mL) and xanthine (50 μg/mL) (86). The epitope tagging of genes was verified by recombination-specific PCR and sequencing of the resultant amplicons. The stable drug-resistant parasites were cloned by limiting dilution in 96-well plates with HFF monolayers. Expression of smHA-tagged P4-ATPases in transgenic strains was regulated by the corresponding native promoter and 3′UTR of HXGPRT.

To perform the 3′-tagging of *TgP*4-ATPase1-3 proteins with a single HA tag, as reported earlier (87), a crossover sequence (1 kb) preceding the stop codon in each gene was amplified from gDNA of the RHΔ*ku80-hxgprt*^*−*^ strain (Pfu Fusion High-Fidelity Polymerase, Agilent Technologies; Table S1). The HA-tagged PCR amplicons of *TgP*4-ATPase1-3 were cloned into the *p3′IT-HXGPRT* plasmid using *Xcm*I and *EcoR*I. This vector harbors a floxed *Tg*Gra2-3′UTR to stabilize the transcript. All 3′-epitope tagging constructs were linearized (20 μg) in the first half of crossover sequence by *Xho*I (*Tg*P4-ATPase1/3) or *EcoR*V (*TgP*4-ATPase2), transfected into tachyzoites of the RHΔ*ku80-hxgprt*^*−*^ strain (10^7^) and selected as described earlier and elsewhere (86). The epitope tagging was confirmed by recombination-specific PCR screening and sequencing of amplicons from the eventual transgenic strains (P_Native_-*Tg*P4-ATPaseX-HA_3′IT_-3′UTR_Gra2 (floxed)_, X = 1 to 3), which expressed HA-tagged proteins regulated by the native promoter and floxed *Tg*Gra2-3′UTR.

### Auxin-inducible knockdown of *TgP*4-ATPase1-3

A knockdown of specified genes was performed by auxin-mediated proteasomal degradation, as reported (59). Gene-specific *sg*RNA-encoding vectors targeting the 3′UTR region located just after the stop codon were prepared, as described earlier (primers in Table S1). They were cotransfected into the RHΔ*ku80*-*hxgprt*^*−*^-TIR1 strain along with the corresponding PCR amplicons, which harbored the AID-3xHA epitope and dihydrofolate reductase–thymidylate synthase (DHFR-TS) selection cassette flanked by 5′ and 3′-homology arms (40 bp) of the respective P4-ATPases. Amplicons were generated using the *pLinker-AID-3xHA-DHFR*-*TS* plasmid template and Phanta Max Super-Fidelity DNA polymerase (Vazyme Biotech) (Table S1). Stable transgenic tachyzoites were selected by 3 μM pyrimethamine, and epitope tagging was ascertained by crossover-specific PCR screening, sequencing, immunofluorescence, and immunoblot assays. The chosen parasite strains were cloned by limiting dilution in 96-well plates and tested for their growth in HFF monolayers by plaque assays in the absence or presence of 500 μM IAA.

### Plaque assay

The experiment was set up with fresh syringe-released parasites, essentially as reported (88). Parasitized cultures (MOI, 2; 40–44 h infection) were washed with standard medium, scraped, and extruded two to three times through a 27-gauge syringe. HFF monolayers grown in six-well plates were infected with tachyzoites (150–200 parasites/well) and incubated for 7 days without any perturbation (37 °C, 5% CO_2_). When indicated, edelfosine and miltefosine (PtdCho analogs; Sigma-Aldrich; 5 mM stock in PBS) were added at the time of initiating plaque cultures (1–100 μM). Following incubation, infected monolayers were fixed with ice-cold methanol (−80 °C, 10 min) and stained with crystal violet (12.5 g dye in 125 mL ethanol mixed with 500 mL of 1% ammonium oxalate) for 15 min, followed by 3× PBS washing. Plaques were enumerated by microscopy, and their size was quantified using the ImageJ software (National Institute of Health).

### Indirect immunofluorescence assay

Confluent host cells grown on the sterile glass coverslips were infected with tachyzoites (MOI, 3; 24–36 h). Cultures were washed with 500 μl PBS, fixed with 4% paraformaldehyde/PBS for 15 min, and then neutralized with 500 μl of glycine solution (0.1 M in PBS, 5 min). Samples were permeabilized with 1 mL of Triton X-100 (0.2% in PBS, 20 min), followed by further incubation for 20 min in 500 μl PBS containing 0.2% Triton X-100, 2% BSA (w/v) to block nonspecific binding. Samples were then treated with 200 μl of primary antibody solution (dissolved in 0.2% Triton X-100, 2% BSA/PBS) for 1 h with repeated gentle swirling. They were washed three times, each for 5 min with 1 mL of 0.2% Triton X-100 in PBS, and afterward incubated with 200 μl of secondary antibody solution for 45 min in dark. Immunostained cultures were washed 3× with PBS and the coverslips were mounted in Fluoromount G and 4′,6-diamidino-2-phenylindole mixture and then stored at 4 °C for 1 h before imaging (Zeiss).

To resolve the C-terminal topology of *Tg*P4-ATPase1, fresh extracellular parasites were stained with mouse α-HA (1:3000) and rabbit α-*Tg*Sag2 (1:1000) antibodies, or with rabbit α-*Tg*Gap45 (1:8000) and mouse α-*Tg*Sag1 (1:10,000) before and after permeabilization, as described elsewhere (89). In brief, parasites (10^5^) were fixed on (0.01%) BSA-coated coverslips using 4% paraformaldehyde and 0.05% glutaraldehyde in PBS (w/v) and then stained as specified earlier for permeabilized samples. In contrast, all solutions were replaced by PBS (*i.e.*, no detergent and BSA) for immunostaining of nonpermeabilized samples. To examine the exact membrane distribution of specific *Tg*P4-ATPases, the IMC was split from the plasmalemma by treating extracellular parasites with α-toxin from *Clostridium septicum* (20 nM, 3 h; List Biological Laboratories), followed by a fixation on 0.01% BSA-coated coverslips and immunostaining, as reported (71).

### Immunoblot analysis

Syringe-released tachyzoites (2 × 10^7^/sample) were harvested from infected cultures and washed by ice-cold PBS (3000*g*, 5 min, 4 °C) to collect the pellets, which were either kept on ice when processing immediately or frozen at –80 °C. Each pellet was resuspended in 50 μl lysis buffer (10 mM K_2_HPO_4_, 150 mM NaCl, 5 mM EDTA, 5 mM EGTA, pH7.4; 0.2% sodium deoxycholate, 1% Triton X-100) and 0.5 μl proteinase inhibitor (trypsin, 20 μg/mL; aprotinin, 10 μg/mL; benzamidine, 500 μg/mL; PMSF, 0.5 mM; Na_3_VO_4_, 0.1 mM; NaF, 5 mM) by syringe squirting and incubated on ice for 15 min, followed by centrifugation (20,000*g*, 15 min, 4 °C) to obtain the cell-free extract. Samples were mixed with 5× loading buffer (2.5 mM DTT and 4% ß-mercaptoethanol), resolved by 6% SDS-PAGE, and blotted onto a nitrocellulose membrane (semidry for 90 min, 90 mA/cm^2^). Immunoblot was probed by α-HA (mouse, 1:10,000) and α-*Tg*Hsp90 (rabbit, 1:10,000) as the primary antibodies (4 °C, overnight) and the corresponding IRDye 680RD and IRDye 800CW (1:15,000) as the secondary antibodies (1 h, room temperature). Protein bands were visualized by Li-COR imaging.

### *In silico* analysis of *Tg*P4-ATPase1-5

The amino acid sequences of *Tg*P4-ATPase1-5 were analyzed using several online tools to predict the primary structure and membrane topology. TMHMM (90), Phobius (91), and TMpred (92) were utilized to discern the number, size, orientation, and location of the transmembrane regions. Helices with a probability score of ≥800 as well as unanimity among the stated software were confirmed by sequence alignment with the human (*Hs*ATP8A1) and yeast (*Sc*Neo1, *Sc*Drs2) P4-ATPases. PFAM v32.0 (93), SMART (94), and NCBI domain search (95) tools were utilized to decrypt the functional domains of *Tg*P4-ATPase1-5 proteins. To identify the signature residues and motifs, *Tg*P4-ATPases were aligned with the human, yeast, and/or apicomplexan P4-ATPases (CLC Genomics Workbench). The regions shown (Figs. S4 and S8) were cropped from the entire alignment and color coded from blue (0%) to red (100%) according to the sequence conservation. The schematic topologies of flippases were generated by the TeXtopo package in the LaTeX typesetting system (96). To build a cladogram, the specified sequences of P4-ATPase homologs were aligned by the Neighborhood Joining method (MegaX, 1000 iterations; Fig. 3*B*). The Jukes–Cantor model was used to test the distance, and a single parsimonious cladogram was visualized by FigTree suite (v1.4.3). Likewise, predicted noncatalytic β subunits (Cdc50/Lem3-like proteins) of apicomplexan parasites were first aligned with their human and yeast orthologs by ClustalW, followed by making of a single parsimonious cladogram (Fig. S7*C*).

### Data presentation and statistics

All assays shown in this study were performed at least three independent times, unless stated otherwise. Figures illustrating images or making of transgenic strains typically show only a representative of the three or more biological replicates. Graphs and statistical significance were generated using the GraphPad Prism (v8) suite. The error bars in graphs signify means with SE from multiple assays. The *p*-values were calculated by Student's *t* test (∗*p* ≤ 0.05; ∗∗*p* ≤ 0.01; ∗∗∗*p* ≤ 0.001).

## Data availability

All original data relevant to this study are included herein. The nucleotide and protein sequences of *Tg*P4-ATPases can be accessed *via* databases as indicated elsewhere in this work.

## Conflict of interest

The authors declare that they have no conflicts of interest with the contents of this article.
