# Supplementary material for: Aminoglycerophospholipid flipping and P4-ATPases in Toxoplasma gondii
Source: J Biol Chem. 2021 Jan 21;296:100315. doi: 10.1016/j.jbc.2021.100315 (PMC7949121; doi:10.1016/j.jbc.2021.100315)
Supplement: Figures S1 to S8 and Table S1 [file mmc1.pdf]

SUPPLEMENT FIGURE LEGENDS

Figure S1: Effect of miltefosine and edelfosine on the lytic cycle of *T. gondii.* Plaque assays using the *RHΔku80-hxgprt−* strain in the absence or presence of drugs (as shown). Crystal violet-stained images show plaques formed by individual tachyzoites, and graphs depict the distribution of their size (arbitrary units or a. u.), as scored by Image J program (150-200 plaques/strain from 3 assays; mean +/- S.E.; **p* ≤ 0.05, ***p* ≤ 0.01).

Figure S2: **Decarboxylation of NBD-PtdSer and ATP depletion in tachyzoites. (A)** NBD-PtdSer decarboxylation by extracellular tachyzoites in the absence or presence of hydroxylamine. Samples were treated with the inhibitor (10 mM) for 30 min before labeling with NBD-PtdSer, lipid extraction and thin layer chromatography (also see Fig 1C). The amount of NBD-PtdSer and NBD-PtdEtn was determined by image analysis. **(B)** TLC demonstrating the presence of PSD activity in the secretome of tachyzoites (RHΔ*ku80-hxgprt*− strain). NBD-PtdSer was incubated either with a secreted fraction or with the source parasites (37°C, 30 min), and lipids were resolved by TLC, as described in *methods*. **(C)** ATP content of extracellular tachyzoites. Parasites (10^7) were incubated in the control or energy-depletion medium and then subjected to ATP measurements by luciferase assay. The bar graphs show the mean values (+/- S.E.) from 3 experiments.

Figure S3: **Membrane topology of P4-ATPase1-5 proteins from *T. gondii***. Predicted topologies of *Tg*P4-ATPase1-5 were modeled using TeXtopo (v1.4). Conserved domains and signature motifs are also highlighted. **TM** (transmembrane helices), **A** (actuator), **P** (phosphorylation), **N** (nucleotide-binding) and **R** (regulatory) domains are color-coded. Only *Tg*P4-ATPase1-2 harbor a well-defined '**R**' domain. The start and end edges of individual domains are putative, and the location of motifs and TM helices is proportionate to the protein length, as predicted.

Figure S4: **Conserved motifs and amino acid residues in *Tg*P4-ATPase1-5 of *T. gondii.*** *Tg*P4-ATPase1-5 proteins were aligned with P4-ATPase domains of *Hs*ATP8A1, *Sc*Drs2, *Tg*ATPase$_P$-GC and/or *Sc*Neo1 to identify the conserved residues and motifs. The alignment was trimmed and color-coded according to their conservation. Boxed amino acids have been experimentally tested for their importance in yeast or human proteins. Accession IDs: *T*gP4-ATPase1, MT268297; *Tg*P4-ATPase2, MT268298; *Tg*P4-ATPase3, MT268299; *Tg*P4-ATPase4, MT268300; *Tg*P4-ATPase5, MT268301; *Tg*ATPase$_P$-GC, TGGT1_254370; *Hs*ATP8A1, Q9Y2Q0; *Sc*Drs2, P39524; *Sc*Neo1, P40527.

Figure S5: **Subcellular location of epitope-tagged *Tg*P4-ATPase1-3 in tachyzoites**. **(A)** Scheme showing the 3'-insertional tagging (3'IT) of *Tg*P4-ATPase1-3 with a C-terminal HA epitope. Linearized plasmids were transfected into the parental RHΔ*ku80-hxgprt*− strain followed by HXGPRT selection. Transgenic parasites expressed HA-tagged *Tg*P4-ATPase1-3 regulated by the native promoter and floxed 3'UTR of *Tg*Gra2. **(B)** Verification of epitope-tagged *Tg*P4-ATPase1-3 by genomic screening

using specific primer pairs (see *panel A*). The *RHΔku80-hxgprt−* strain served as a negative control. **(C)** Tachyzoites encoding for *Tg*P4-ATPaseX-HA$_{3'IT}$ (X=1-3) were stained with α-HA and α-*Tg*Gap45 antibodies and DAPI. Scale bars, 2 μm. *COS*, crossover sequence; *S.C.*, selection cassette.

Figure S6: Genomic tagging of *Tg*P4-ATPase1-5 proteins with spaghetti monster epitope does not affect the tachyzoite growth. Plaques formed by smHA-tagged strains (P$_{native}$-*Tg*P4-ATPaseX-smHA$_{3'IT}$-3'UTR$_{Gra1}$, X= 1-5) in comparison to the parental strain. The plaque area (arbitrary units or a. u.), as measured by ImageJ, is shown (150-200 plaques/strain; n = 3 assays; means +/- S.E.).

Figure S7: Localization of *Tg*Lem3 in the Golgi network of *T. gondii* and its phylogeny. (A) Intracellular tachyzoites expressing *Tg*Lem3-HA. The Lem3/Cdc50 homolog (TGGT1_239540) was cloned in a *pTETO7SAG1-UPKO* plasmid (primers in Table S1), and its expression was driven by the p*TETO7SAG1* promoter and *Tg*Sag1-3'UTR. The Lem3 expression cassette was targeted at the uracil phosphoribosyltransferase (*UPRT*) locus by double homologous recombination *via* negative selection (5 μM 5-fluorodeoxyuridine) in the RHΔ*ku80-hxgprt−* strain. Immunostaining of tachyzoites was performed 24 h post-infection using α-HA/Alexa488 and α-*Tg*Gap45/Alexa594 antibodies. **(B)** Co-localization of *Tg*Lem3-HA and *Tg*Erd2-Ty1 in tachyzoites. Parasites expressing Lem3-HA from *panel A* were transfected with the *pTUB8-TgErd2-Ty1* construct and selected (1 μM pyrimethamine) for the expression of a drug-resistant dihydrofolate reductase – thymidylate synthase (DHFR-TS). Transgenic tachyzoites harboring both expression cassettes (E.C.) were immunostained using rabbit α-HA and α-*Tg*Gap45, and mouse α-Ty1 antibodies (30 h infection). **(C)** Phylogenetic clading of the predicted apicomplexan Lem3/Cdc50 proteins with their yeast and human orthologs. The amino acid sequences of specified β-subunits were aligned and clustered using the ClustalW and MegaX suites. EuPathDB accession numbers of apicomplexan proteins are shown next to the cladogram: TGGT1, *T. gondii* GT1 strain; ETH, *E. tenella* Houghton strain; PF3DF, *P. falciparum* 3D7 strain; CPATCC, *C. parvum* ATCC strain. NCBI accession IDs of human and yeast proteins: *Hs*Cdc50A, NP_060717.1; *Hs*Cdc50B, NP_001017970.1; *Hs*Cdc50C, A0ZSE6.2; *Sc*Cdc50, NP_010018.1; *Sc*Lem3, NP_014076.1; *Sc*CRF1, NP_014446.3

Figure S8: Alignment of conserved motifs in *Tg*P4-ATPase1 and its nearest orthologs in other apicomplexan parasites. The *Tg*P4-ATPase1 sequence was aligned with P4-ATPase domains of *Pf*P4-ATPase1, *Et*P4-ATPase1, *Cp*P4-ATPase1, followed by sequence trimming and color-coding according to conservation. EuPathDB accession: *T*gP4-ATPase1, TGGT1_247690; *Pf*P4-ATPase1, PF3D7_1219600; *Et*P4-ATPase1, ETH_00018890; *Cp*P4-ATPase1, CPATCC_0033740

Figure S1

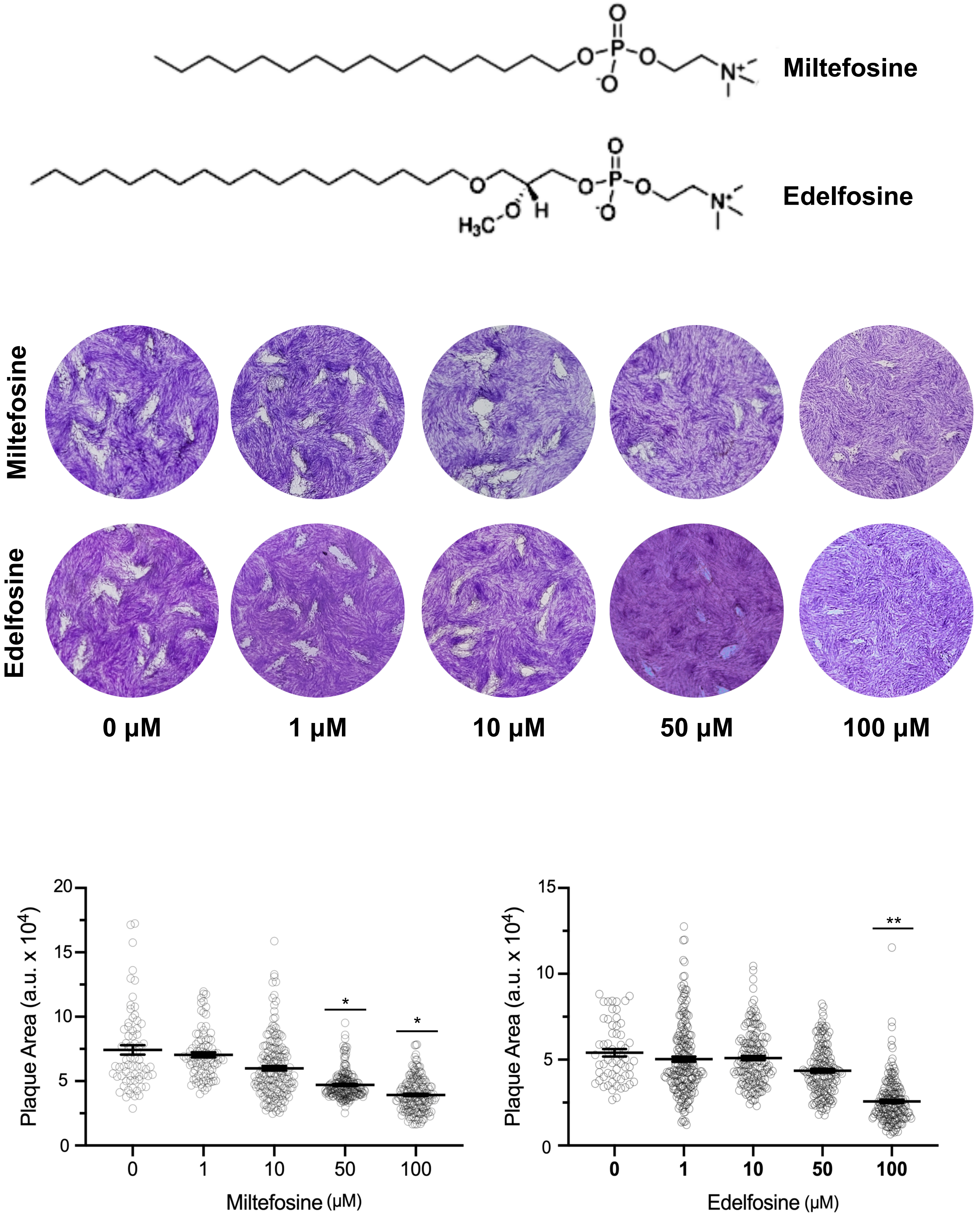

Miltefosine
Edelfosine
H3C
Miltefosine
Edelfosine
0 µM
1 µM
10 µM
50 µM
100 µM
Plaque Area (a.u. x 10⁴)
20
15
10
5
0
0
1
10
50
100
*
*
Miltefosine (µM)
Plaque Area (a.u. x 10⁴)
15
10
5
0
0
1
10
50
100
**
Edelfosine (µM)

Figure S2

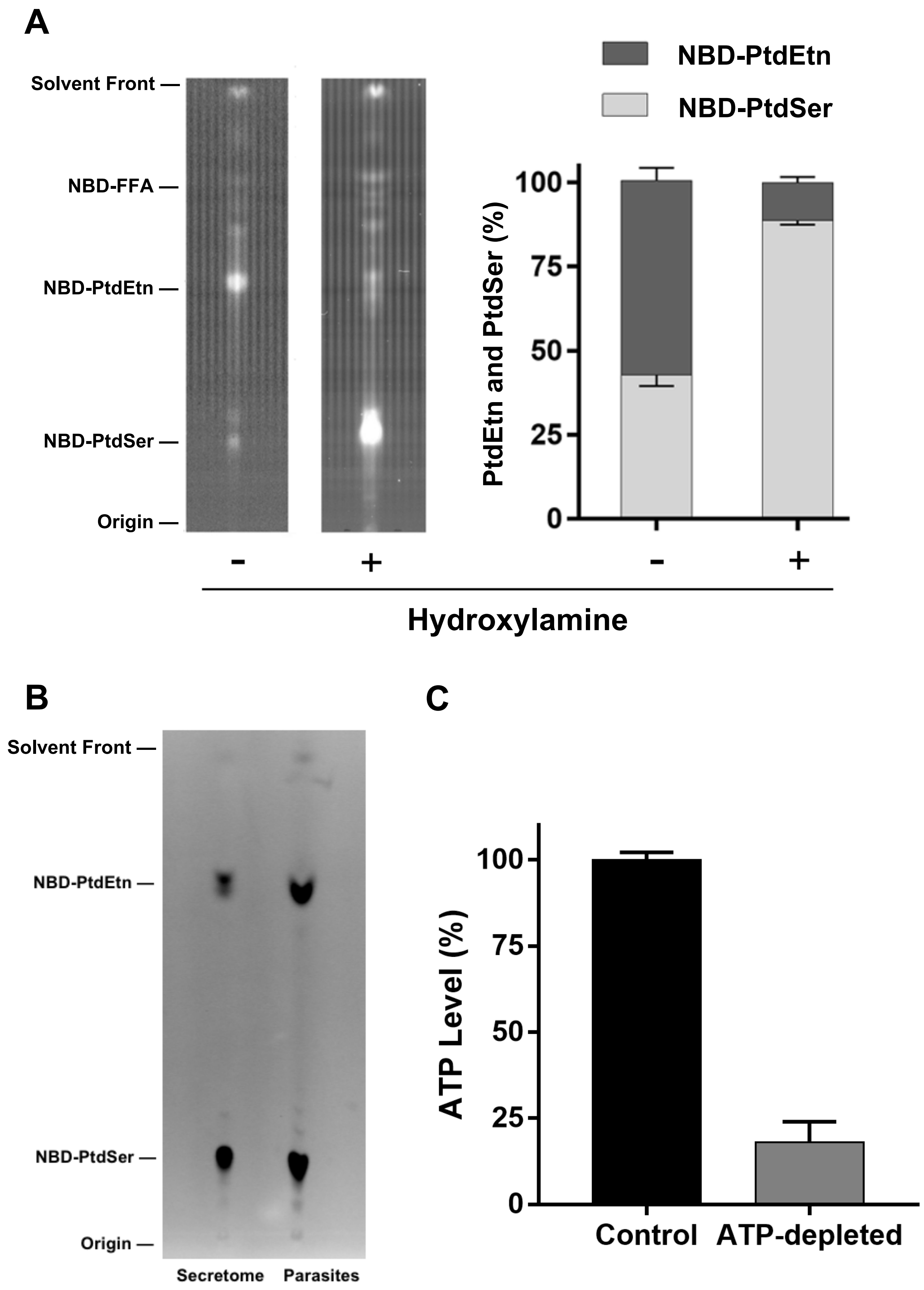
A
Solvent Front
NBD-FFA
NBD-PtdEtn
NBD-PtdSer
Origin
NBD-PtdEtn
NBD-PtdSer
PtdEtn and PtdSer (%)
100
75
50
25
0
-
+
-
+
Hydroxylamine
B
Solvent Front
NBD-PtdEtn
NBD-PtdSer
Origin
Secretome
Parasites
C
ATP Level (%)
100
75
50
25
0
Control
ATP-depleted

Figure S3

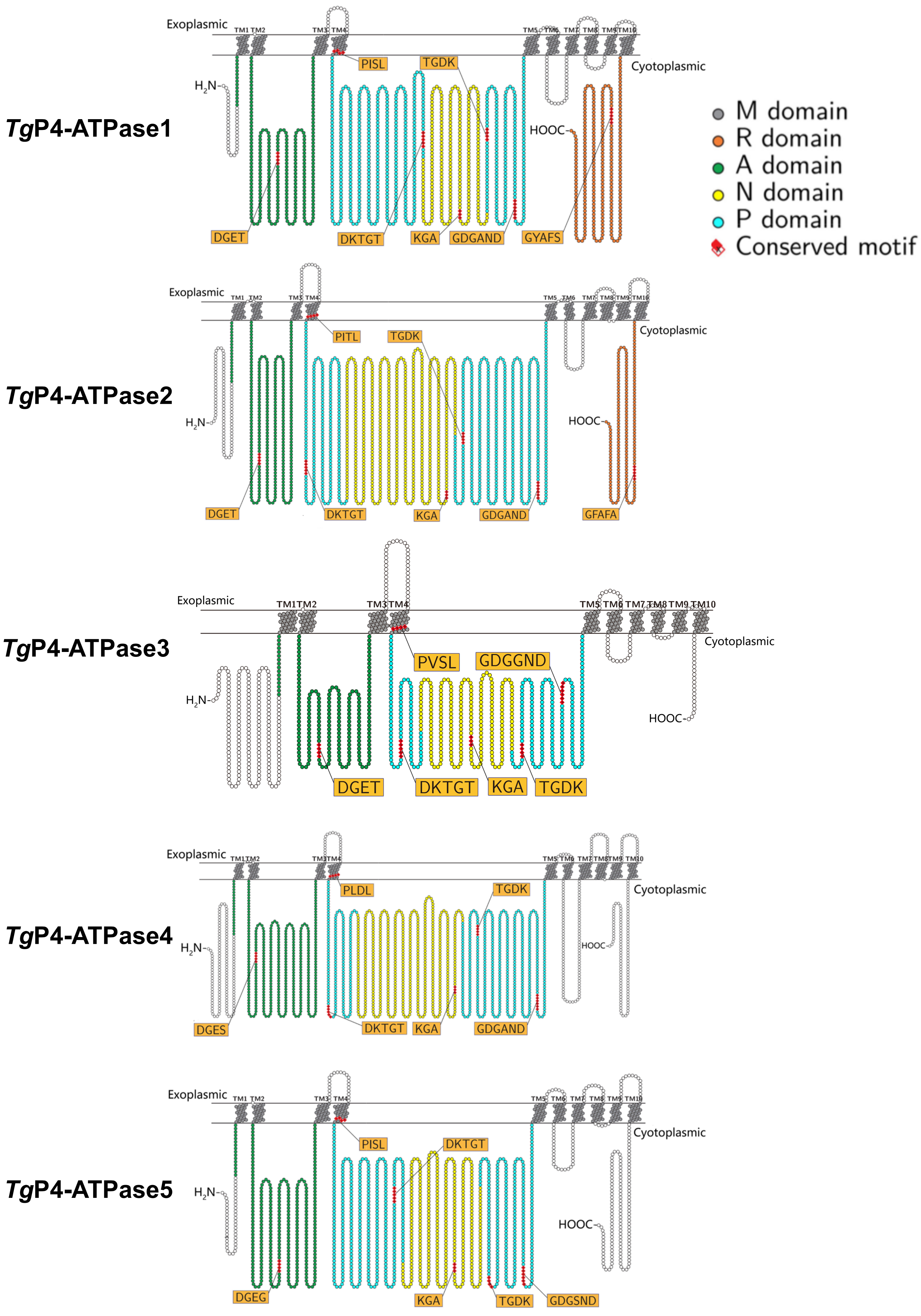
TgP4-ATPase1
Exoplasmic
TM1 TM2
TM3 TM4
TM5 TM6 TM7 TM8 TM9 TM10
Cytoplasmic
H2N-
HOOC-
PISL
TGDK
DGET
DKTGT
KGA
GDGAND
GYAFS
M domain
R domain
A domain
N domain
P domain
Conserved motif
TgP4-ATPase2
Exoplasmic
TM1 TM2
TM3 TM4
TM5 TM6 TM7 TM8 TM9 TM10
Cytoplasmic
H2N-
HOOC-
PITL
TGDK
DGET
DKTGT
KGA
GDGAND
GFAFA
TgP4-ATPase3
Exoplasmic
TM1TM2
TM3TM4
TM5 TM6 TM7 TM8 TM9 TM10
Cytoplasmic
H2N-
HOOC-
PVSL
GDGGND
DGET
DKTGT
KGA
TGDK
TgP4-ATPase4
Exoplasmic
TM1TM2
TM3TM4
TM5 TM6 TM7 TM8 TM9 TM10
Cytoplasmic
H2N-
HOOC-
PLDL
TGDK
DGES
DKTGT
KGA
GDGAND
TgP4-ATPase5
Exoplasmic
TM1 TM2
TM3 TM4
TM5 TM6 TM7 TM8 TM9 TM10
Cytoplasmic
H2N-
HOOC-
PISL
DKTGT
DGEG
KGA
TGDK
GDGSND

Figure S4

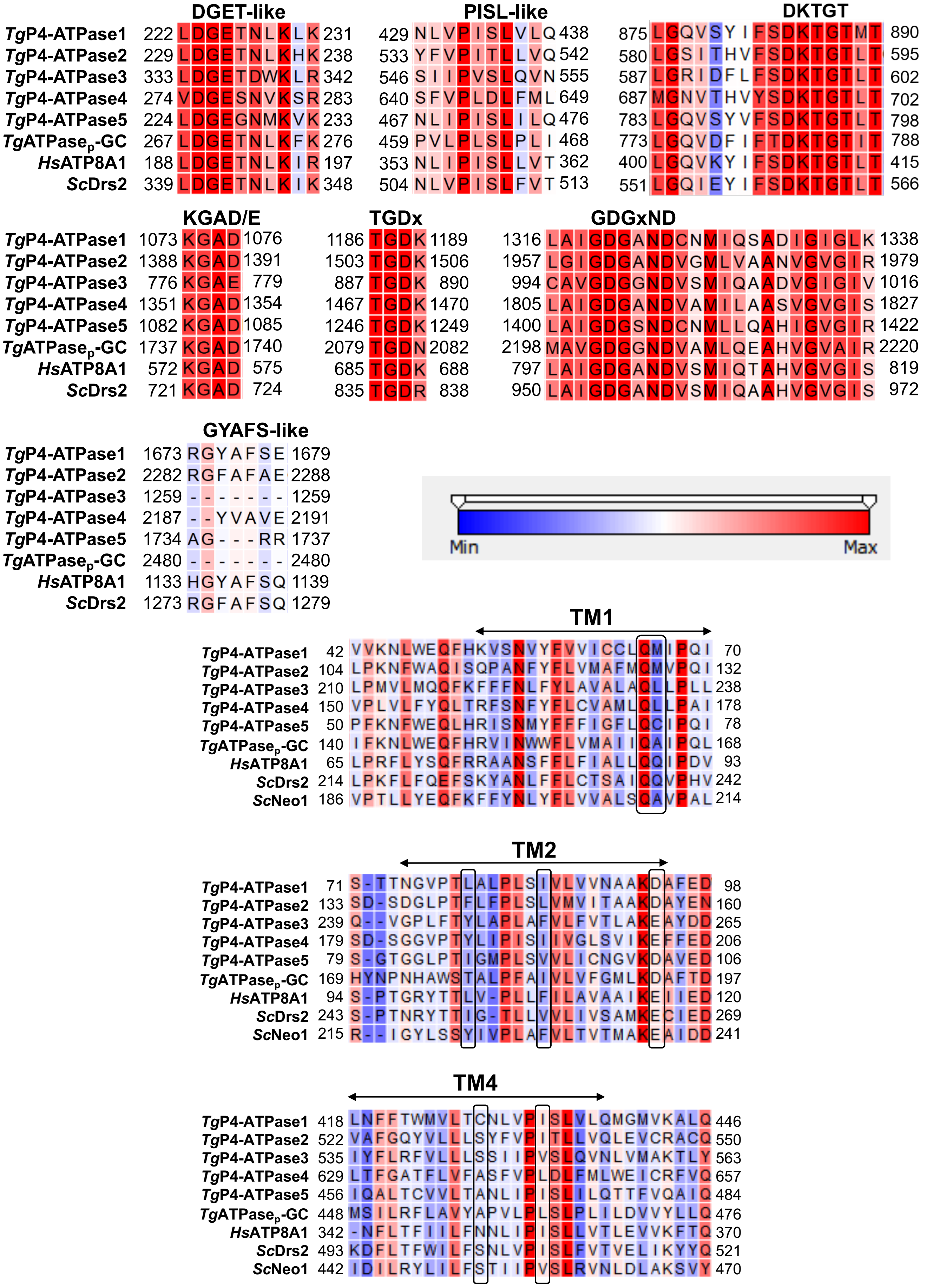
DGET-like
TgP4-ATPase1 222 LDGETNLKLK 231
TgP4-ATPase2 229 LDGETNLKHK 238
TgP4-ATPase3 333 LDGETDWKLR 342
TgP4-ATPase4 274 VDGESNVKSR 283
TgP4-ATPase5 224 LDGEGNMKVK 233
TgATPasep-GC 267 LDGETNLKFK 276
HsATP8A1 188 LDGETNLKIR 197
ScDrs2 339 LDGETNLKIK 348
PISL-like
429 NLVPISLVLQ 438
533 YFVPITLLVQ 542
546 SIIPVSLQVN 555
640 SFVPLDLFML 649
467 NLIPISLILQ 476
459 PVLPLSLPLI 468
353 NLIPISLLVT 362
504 NLVPISLFVT 513
DKTGT
875 LGQVSYIFSDKTGTMT 890
580 LGSITHVFSDKTGTLT 595
587 LGRIDFLFSDKTGTLT 602
687 MGNVTHVYSDKTGTLT 702
783 LGQVSYVFSDKTGTLT 798
773 LGQVDFIFTDKTGTIT 788
400 LGQVKYIFSDKTGTLT 415
551 LGQIEYIFSDKTGTLT 566
KGAD/E
TgP4-ATPase1 1073 KGAD 1076
TgP4-ATPase2 1388 KGAD 1391
TgP4-ATPase3 776 KGAE 779
TgP4-ATPase4 1351 KGAD 1354
TgP4-ATPase5 1082 KGAD 1085
TgATPasep-GC 1737 KGAD 1740
HsATP8A1 572 KGAD 575
ScDrs2 721 KGAD 724
TGDx
1186 TGDK 1189
1503 TGDK 1506
887 TGDK 890
1467 TGDK 1470
1246 TGDK 1249
2079 TGDN 2082
685 TGDK 688
835 TGDR 838
GDGxND
1316 LAIGDGANDCNMIQSADIGIGLK 1338
1957 LGIGDGANDVGMLVAANVGVGIR 1979
994 CAVGDGGNDVSMIQAADVGIGIV 1016
1805 LAIGDGANDVAMILAASVGVGIS 1827
1400 LAIGDGSNDCNMLLQAHIGVGIR 1422
2198 MAVGDGGNDVAMLQEAHVGVAIR 2220
797 LAIGDGANDVSMIQTAHVGVGIS 819
950 LAIGDGANDVSMIQAAHVGVGIS 972
GYAFS-like
TgP4-ATPase1 1673 RGYAFSE 1679
TgP4-ATPase2 2282 RGFAFAE 2288
TgP4-ATPase3 1259 - - - - - - - 1259
TgP4-ATPase4 2187 - - YVAVE 2191
TgP4-ATPase5 1734 AG - - - RR 1737
TgATPasep-GC 2480 - - - - - - - 2480
HsATP8A1 1133 HGYAFSQ 1139
ScDrs2 1273 RGFAFSQ 1279
Min
Max
TM1
TgP4-ATPase1 42 VVKNLWEQFHKVSNVYFVVICCLQMIPQI 70
TgP4-ATPase2 104 LPKNFWAQISQPANFYFLVMAFMQMVPQI 132
TgP4-ATPase3 210 LPMVLMQQFKFFFNLFYLAVALAQLLPLL 238
TgP4-ATPase4 150 VPLVLFYQLTRFSNFYFLCVAMLQLLPAI 178
TgP4-ATPase5 50 PFKNFWEQLHRISNMYFFFIGFLQCIPQI 78
TgATPasep-GC 140 IFKNLWEQFHRVINWWFLVMAIIQAIPQL 168
HsATP8A1 65 LPRFLYSQFRRAANSFFLFIALLQQIPDV 93
ScDrs2 214 LPKFLFQEFSKYANLFFLCTSAIQQVPHV 242
ScNeo1 186 VPTLLYEQFKFFYNLYFLVVALSQAVPAL 214
TM2
TgP4-ATPase1 71 S-TTNGVPTLALPLSIVLVVNAAKDAFED 98
TgP4-ATPase2 133 SD-SDGLPTFLFPLSLVMVITAAKDAYEN 160
TgP4-ATPase3 239 Q--VGPLFTYLAPLAFVLFVTLAKEAYDD 265
TgP4-ATPase4 179 SD-SGGVPTYLIPISIIVGLSVIKEFFED 206
TgP4-ATPase5 79 S-GTGGLPTIGMPLSVVLICNGVKDAVED 106
TgATPasep-GC 169 HYNPNHAWSTALPFAIVLVFGMLKDAFTD 197
HsATP8A1 94 S-PTGRYTTLV-PLLFILAVAAIKEIIED 120
ScDrs2 243 S-PTNRYTTIG-TLLVVLIVSAMKECIED 269
ScNeo1 215 R--IGYLSSYIVPLAFVLTVTMAKEAIDD 241
TM4
TgP4-ATPase1 418 LNFFTWMVLTCNLVPISLVLQMGMVKALQ 446
TgP4-ATPase2 522 VAFGQYVLLLSYFVPITLLVQLEVCRACQ 550
TgP4-ATPase3 535 IYFLRFVLLLSSIIPVSLQVNLVMAKTLY 563
TgP4-ATPase4 629 LTFGATFLVFASFVPLDLFMLWEICRFVQ 657
TgP4-ATPase5 456 IQALTCVVLTANLIPISLILQTTFVQAIQ 484
TgATPasep-GC 448 MSILRFLAVYAPVLPLSLPLILDVVYLLQ 476
HsATP8A1 342 -NFLTFIILFNNLIPISLLVTLEVVKFTQ 370
ScDrs2 493 KDFLTFWILFSNLVPISLFVTVELIKYYQ 521
ScNeo1 442 IDILRYLILFSTIIPVSLRVNLDLAKSVY 470

Figure S5

A

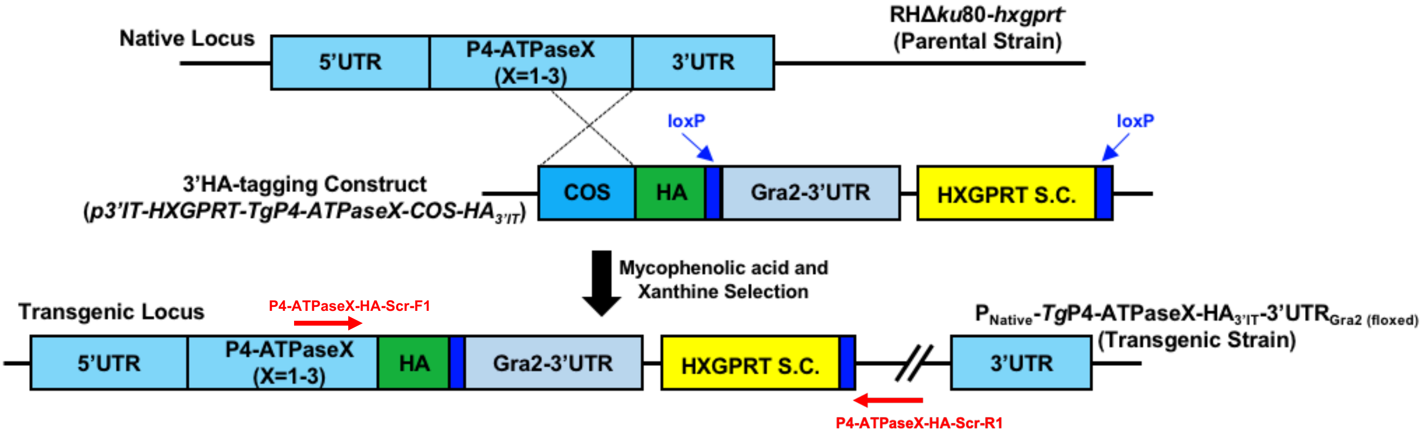

B

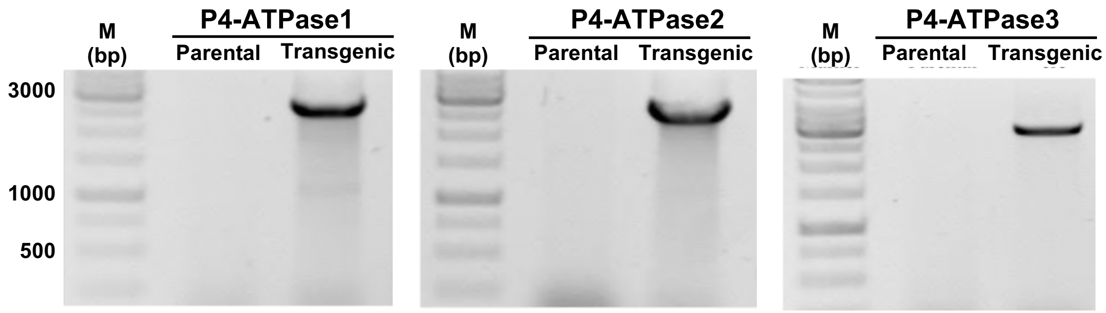

C

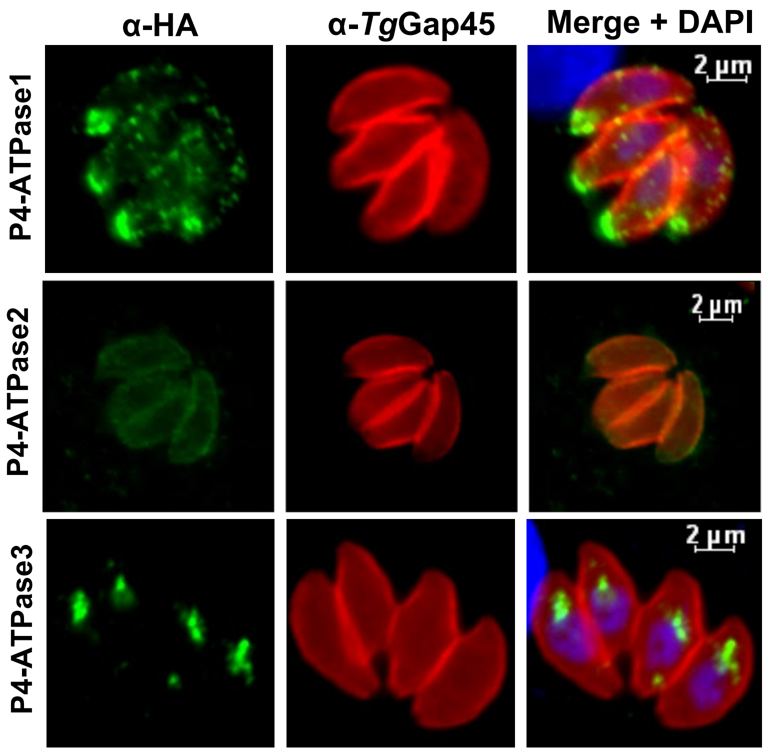

Figure S6

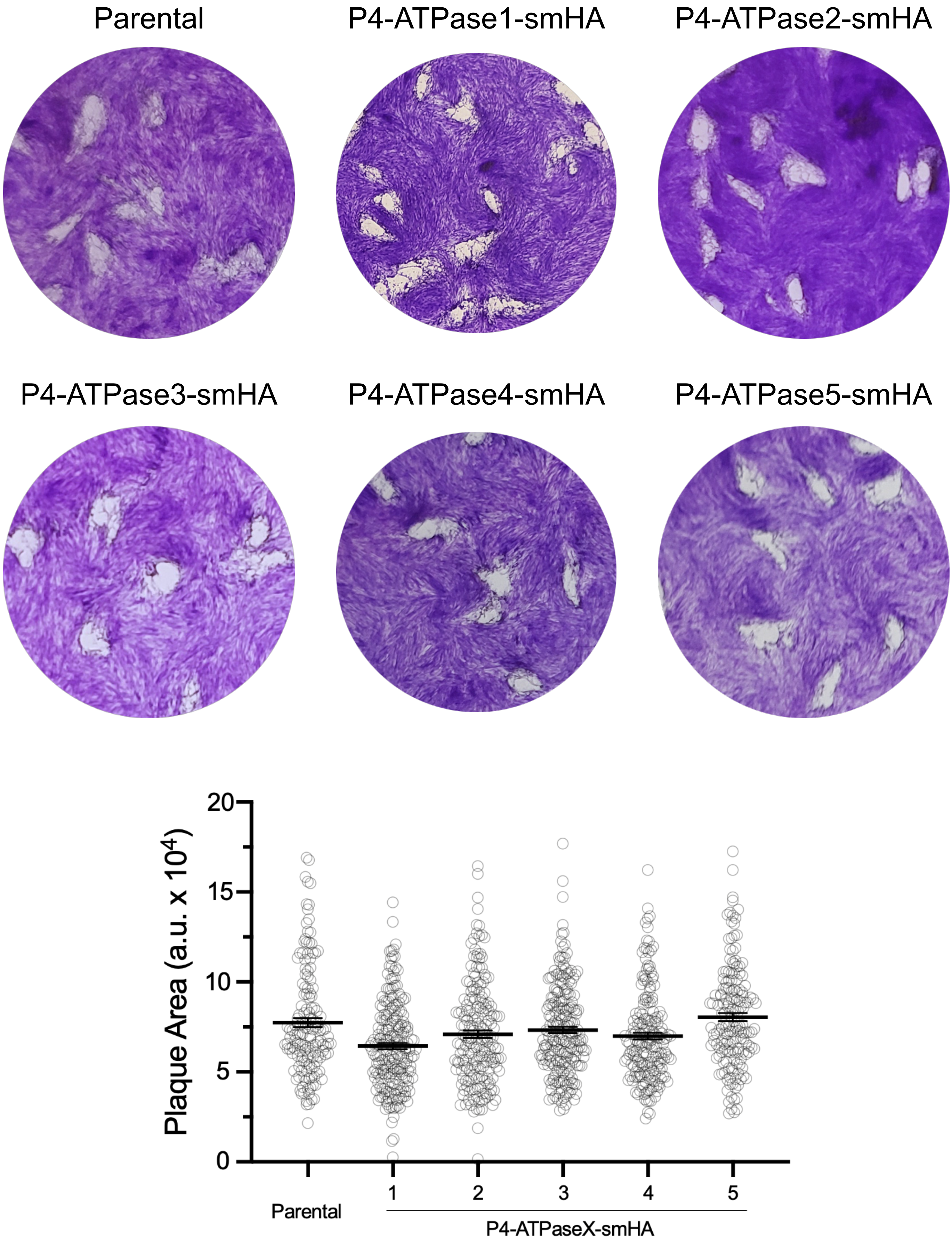
Parental
P4-ATPase1-smHA
P4-ATPase2-smHA
P4-ATPase3-smHA
P4-ATPase4-smHA
P4-ATPase5-smHA
Plaque Area (a.u. x 10⁴)
20
15
10
5
0
Parental
1
2
3
4
5
P4-ATPaseX-smHA

Figure S7

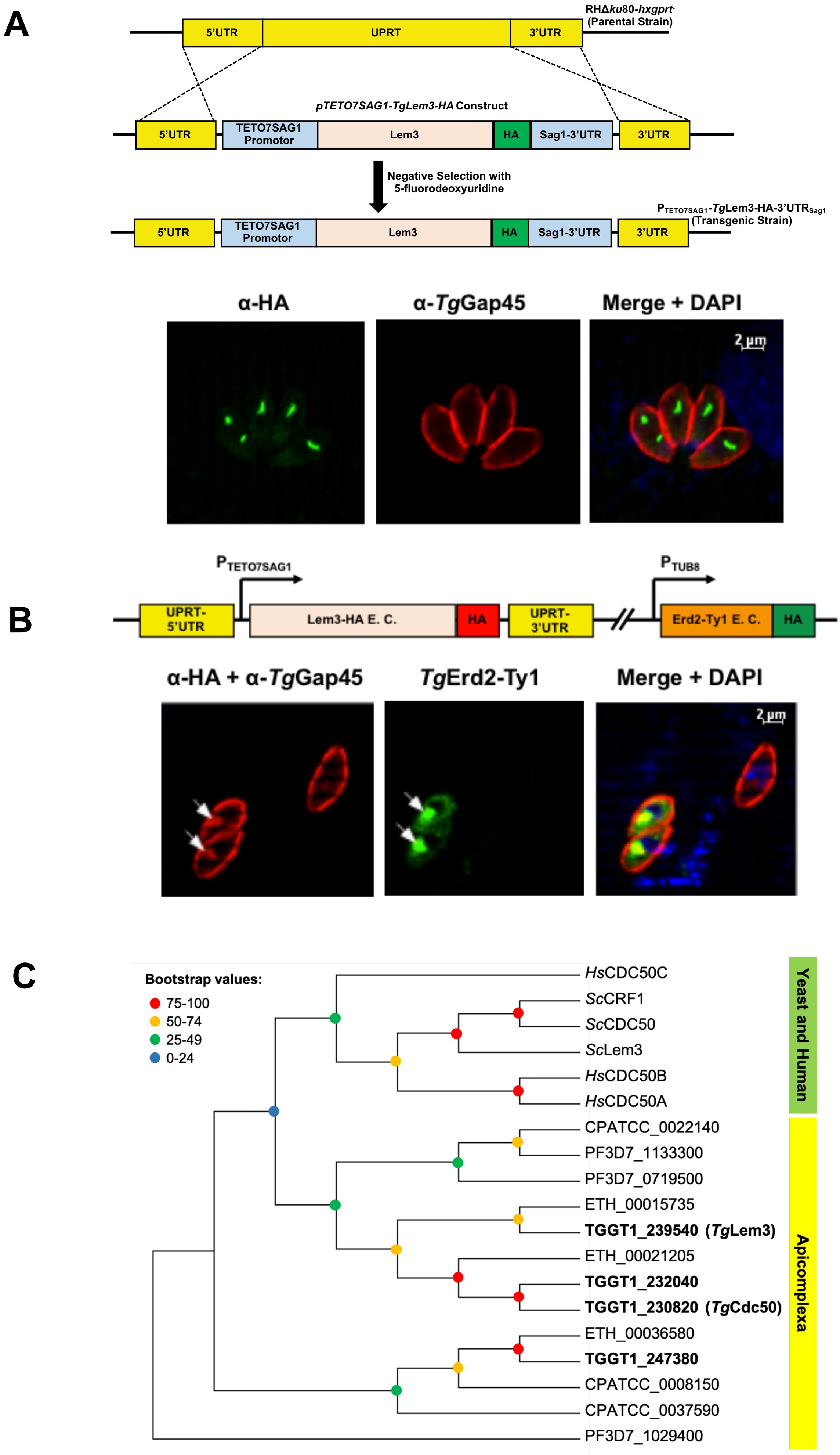
A
5'UTR
UPRT
3'UTR
RHΔku80-hxgprt
(Parental Strain)
pTETO7SAG1-TgLem3-HA Construct
TETO7SAG1 Promotor
Lem3
HA
Sag1-3'UTR
Negative Selection with 5-fluorodeoxyuridine
PTETO7SAG1-TgLem3-HA-3'UTRSag1
(Transgenic Strain)
α-HA
α-TgGap45
Merge + DAPI
2 μm
B
PTETO7SAG1
PTUB8
UPRT-5'UTR
Lem3-HA E. C.
UPRT-3'UTR
Erd2-Ty1 E. C.
α-HA + α-TgGap45
TgErd2-Ty1
C
Bootstrap values:
75-100
50-74
25-49
0-24
HsCDC50C
ScCRF1
ScCDC50
ScLem3
HsCDC50B
HsCDC50A
CPATCC_0022140
PF3D7_1133300
PF3D7_0719500
ETH_00015735
TGGT1_239540 (TgLem3)
ETH_00021205
TGGT1_232040
TGGT1_230820 (TgCdc50)
ETH_00036580
TGGT1_247380
CPATCC_0008150
CPATCC_0037590
PF3D7_1029400
Yeast and Human
Apicomplexa

Figure S8

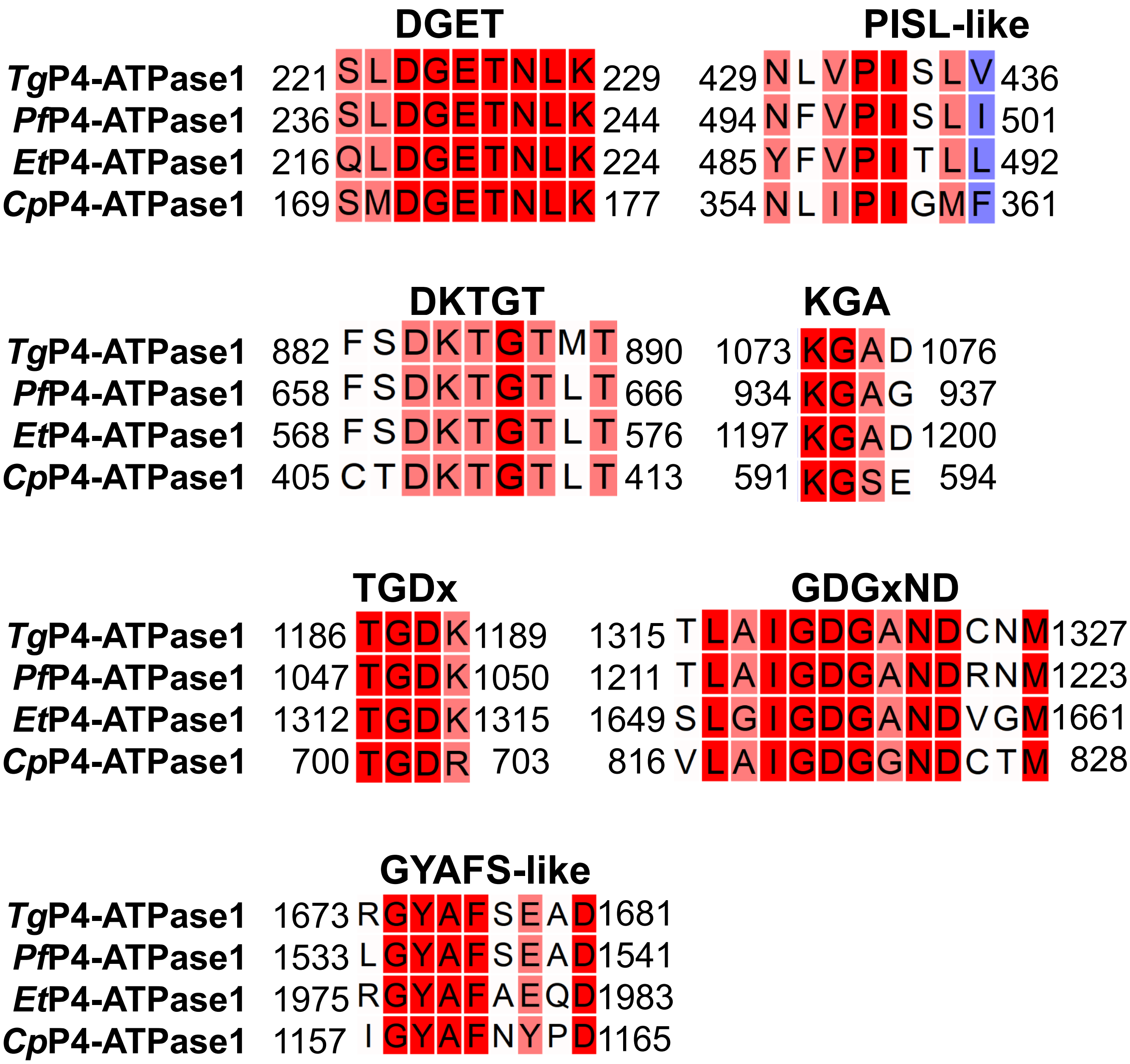
DGET
PISL-like
TgP4-ATPase1 221 SLDGETNLK 229 429 NLVPISLV 436
PfP4-ATPase1 236 SLDGETNLK 244 494 NFVPISLI 501
EtP4-ATPase1 216 QLDGETNLK 224 485 YFVPITLL 492
CpP4-ATPase1 169 SMDGETNLK 177 354 NLIPIGMF 361
DKTGT
KGA
TgP4-ATPase1 882 FSDKTGTMT 890 1073 KGAD 1076
PfP4-ATPase1 658 FSDKTGTLT 666 934 KGAG 937
EtP4-ATPase1 568 FSDKTGTLT 576 1197 KGAD 1200
CpP4-ATPase1 405 CTDKTGTLT 413 591 KGSE 594
TGDx
GDGxND
TgP4-ATPase1 1186 TGDK 1189 1315 TLAIGDGANDCNM 1327
PfP4-ATPase1 1047 TGDK 1050 1211 TLAIGDGANDRNM 1223
EtP4-ATPase1 1312 TGDK 1315 1649 SLGIGDGANDVGM 1661
CpP4-ATPase1 700 TGDR 703 816 VLAIGDGGNDCTM 828
GYAFS-like
TgP4-ATPase1 1673 RGYAFSEAD 1681
PfP4-ATPase1 1533 LGYAFSEAD 1541
EtP4-ATPase1 1975 RGYAFAEQD 1983
CpP4-ATPase1 1157 IGYAFNYPD 1165

Table S1: Oligonucleotides, constructs and parasite strains used in this study

Primer Name (restriction site)	Nucleotide Sequence (*sg*RNA, restriction site or homology arms are underlined)	Cloning Vector (research objective)
**CRISPR/Cas9-assisted 3'-insertional tagging (3'IT) of *Tg*P4-ATPaseX (X = 1-5) genes with a smHA epitope in the RHΔ*ku80-hxgprt*⁻strain**		
*Tg*P4-ATPase1-*sg*RNA-3'IT-F1	GACAGTGCAGAAGGATGAGCGTTTTAGAGCTAGAAATAGC	***pU6-Cas9*** (CRISPR *sg*RNA construction)
*Tg*P4-ATPase2-*sg*RNA-3'IT-F1	GGCACGCAGAAATAGCGCTCGTTTTAGAGCTAGAAATAGC	
*Tg*P4-ATPase3-*sg*RNA-3'IT-F1	GGAGACAAAGGACGGTTCAGGTTTTAGAGCTAGAAATAGC	
*Tg*P4-ATPase4-*sg*RNA-3'IT-F1	GCGTGCTATGCGAAATCAGCGTTTTAGAGCTAGAAATAGC	
*Tg*P4-ATPase5-*sg*RNA-3'IT-F1	GCAGGTAGCAGGTTTCTGTAGTTTTAGAGCTAGAAATAGC	
*Tg*P4-ATPaseX-*sg*RNA-3'IT-R1	AACTTGACATCCCCATTTAC	
*Tg*P4-ATPase1-smHA-COS-F1	GGAGTGTTACAGGCATCGTGTGCAGCAGTCCAGAGGATTGAATTCCCTAGGATGTACCCT	***pLIC-smHA-HXGPRT*** (PCR of smHA and HXGPRT selection cassette flanked by 5' and 3' homology arms)
*Tg*P4-ATPase1-smHA-COS-R1	TAATTTCGCAGAAGGCTTCCCCGCTCCATGGCTCTTCGCAATAGGGCGAATTGGAGCTCC	
*Tg*P4-ATPase2-smHA-COS-F1	CCCCGGAGCTTCCGCGCCGCGGAACGCAGCCAACGATGCGAATTCCCTAGGATGTACCCT	
*Tg*P4-ATPase2-smHA-COS-R1	ATTCACTTACATACCCTGAGATACATGTACGTATATATATATAGGGCGAATTGGAGCTCC	
*Tg*P4-ATPase3-smHA-COS-F1	GACCCTCCAGCCGCCTCAGCATGCGAAGCTCGCGTCGCCGAATTCCCTAGGATGTACCCT	
*Tg*P4-ATPase3-smHA-COS-R1	GTCTCGTGTCCAGAGAACCACAAGGAAATACAGAAACAGAATAGGGCGAATTGGAGCTCC	
*Tg*P4-ATPase4-smHA-COS-F1	GACATTAATTAAAGGTGGCGAGGGGAGTGATGCGAAGAAAGAATTCCCTAGGATGTACCCT	
*Tg*P4-ATPase4-smHA-COS-R1	AAATATTCCAACACCAGGAAGCCCACTATGAATGAATTTCATAGGGCGAATTGGAGCTCC	
*Tg*P4-ATPase5-smHA-COS-F1	GGCGCCGATTCTTCTCAACCTGCAGAGATCCTTGAGCCTTGAATTCCCTAGGATGTACCCT	
*Tg*P4-ATPase5-smHA-COS-R1	TTTCTCCATTATTACGGTCTCGGTAGAATTCTCGAAAAAGATAGGGCGAATTGGAGCTCC	
*Tg*P4-ATPase1-smHA-Scr-F1	CGTAGATCCGCATGACAG	***pDrive*** (Screening PCR for verification of smHA tagging)
*Tg*P4-ATPase2-smHA-Scr-F1	GTTCCGTCCTTCCTACTC	
*Tg*P4-ATPase3-smHA-Scr-F1	TTCTGAGACCGAAGCAAAG	
*Tg*P4-ATPase4-smHA-Scr-F1	GAAGGTGTGGCGTATGTT	
*Tg*P4-ATPase5-smHA-Scr-F1	TTCCATCGTCTTGTGTCTC	
*Tg*P4-ATPaseX-smHA-Scr-R1	GGCTGTTGAAGTTGTATTCC	
**3'-insertional tagging (3'IT) of *Tg*P4-ATPaseX (X = 1-3) genes with an AID-3xHA motif in the RHΔ*ku80-hxgprt*⁻- TIR1 strain**		
*Tg*P4-ATPase1-AID-COS-F1	GGAGTGTTACAGGCATCGTGTGCAGCAGTCCAGAGGATTGATGGGCAGTGTCGAGCTG	***pLinker-AID-3xHA-DHFR-TS*** (PCR of AID-3xHA and DHFR-TS selection cassette flanked by 5' and 3' homology arms)
*Tg*P4-ATPase1-AID-COS-R1	TAATTTCGCAGAAGGCTTCCCCGCTCCATGGCTCTTCGCATGTCACTGTAGCCTGCCAGA	
*Tg*P4-ATPase2-AID-COS-F1	CCCCGGAGCTTCCGCGCCGCGGAACGCAGCCAACGATGCGATGGGCAGTGTCGAGCTG	
*Tg*P4-ATPase2-AID-COS-R1	ATATATATACGTACATGTATCTCAGGGTATGTAAGTGAATTGTCACTGTAGCCTGCCAGA	
*Tg*P4-ATPase3-AID-COS-F1	GACCCTCCAGCCGCCTCAGCATGCGAAGCTCGCGTCGCCGATGGGCAGTGTCGAGCTG	
*Tg*P4-ATPase3-AID-COS-R1	GTCTCGTGTCCAGAGAACCACAAGGAAATACAGAAACAGATGTCACTGTAGCCTGCCAGA	
*Tg*P4-ATPase1-AID-Scr-F1	CGTAGATCCGCATGACAG	***pDrive*** (Screening PCR for verification of AID-3xHA tagging)
*Tg*P4-ATPase2-AID-Scr-F1	GTTCCGTCCTTCCTACTC	
*Tg*P4-ATPase3-AID-Scr-F1	TTCTGAGACCGAAGCAAAG	
*Tg*P4-ATPaseX-AID-Scr-R1	GTCTATCATTCCTTCTTCTCCT	
**3'-insertional tagging (3'IT) of *Tg*P4-ATPaseX (X = 1-3) genes with 1x HA epitope in the RHΔ*ku80-hxgprt*⁻strain**		
*Tg*P4-ATPase1-HA-COS-F1 (*Xcm*I)	CTCATCCCACCGGTCACCTGGACGCGTGGCTACGCCTTC	***pTKO-HXGPRT*** (3'-HA-tagging of P4-ATPases)
*Tg*P4-ATPase1-HA-COS-R1 (*EcoR*I)	CTCATCGAATTCCTACGCGTAGTCCGGGACGTCGTACGGGTACAATCCTCTGGACTGCTGCAC	
*Tg*P4-ATPase2-HA-COS-F1 (*Xcm*I)	CTCATCCCACCGGTCACCTGGGCCATCATGTGA GAGGGTATTGATCTT	
*Tg*P4-ATPase2-HA-COS-R1 (*EcoR*I)	CTCATCGAATTCCTACTACGCGTAGTCCGGGA CGTCGTACGGGTACGCATCGTTGGCTGCGTT	
*Tg*P4-ATPase3-HA-COS-F1 (*Xcm*I)	CTCATCCCACCGGTCACCTGGTCTTCCCTCTCTC TTCTGAGTCTCTCG	
*Tg*P4-ATPase3-HA-COS-R1 (*EcoR*I)	CTCATCGAATTCCTACGCGTAGTCCGGGACGT CGTACGGGTACGGCGACGCGAGCTTC	
*Tg*P4-ATPase1-HA-Scr-F1	CTCTAGAAAGCCGCGTGAGG	***pDrive*** (PCR screening to confirm the 3'-HA tagging of P4-ATPases)
*Tg*P4-ATPase2-HA-Scr-F1	GTTTGAGCAGGGCAGCTTG	
*Tg*P4-ATPase3-HA-Scr-F1	GAGGAGCGCAGAAAGGAGAG	
*Tg*P4-ATPaseX-HA-Scr-R1	CAATTTCACACAGGAAACAGCTAT	
**Ectopic expression of *Tg*Lem3 in the RHΔ*ku80*-TaTi strain**		
*Tg*Lem3-F1 (*EcoR*V)	CTCATCGATATCATGCTTCGTCAAAATCGGAG	***pTETO7SAG1-UPKO*** (*Tg*Lem3 expression, regulated by TETO7SAG1 and Sag1-3'UTR, at *UPRT* locus)
*Tg*Lem3-HA-R1 (*Pac*I)	CTCATCTTAATTAATCAAGCGTAATCTGGAACATCGTATGGGTAACTGCCCGCGTAAAGAGC	
